# Conflict misperceptions between citizens and foreigners across the globe

**DOI:** 10.1093/pnasnexus/pgac267

**Published:** 2022-11-22

**Authors:** Angelo Romano, Jörg Gross, Carsten K W De Dreu

**Affiliations:** Social, Economic, and Organizational Psychology Department, Leiden University, 2300 RA Leiden, The Netherlands; Social, Economic, and Organizational Psychology Department, Leiden University, 2300 RA Leiden, The Netherlands; Department of Psychology, University of Zurich, 8050 Zurich, Switzerland; Social, Economic, and Organizational Psychology Department, Leiden University, 2300 RA Leiden, The Netherlands; Center for Experimental Economics and Political Decision Making (CREED), University of Amsterdam, 1001 NJ Amsterdam, The Netherlands

**Keywords:** conflict, cooperation, misperceptions, cross-cultural, ecology

## Abstract

In a globalizing world, conflict between citizens and foreigners hinders cooperation and hampers how well the global community can tackle shared problems. Here, we study conflict between citizens and foreigners and find that people substantially misperceive how competitive foreigners are. Citizens (from 51 countries; *N* = 12,863; 656,274 decisions) interacted with foreigners in incentivized contest experiments. People across the globe systematically failed to anticipate the competitiveness of foreigners and either competed too much or too little. Competition was poorly explained by differences in cultural values or environmental stress. By contrast, competition and concomitant conflict misperceptions were robustly accounted for by differences in the wealth of nations, institutions, and histories of engaging in international conflict. Our results reveal how macro-level socio-economic differences between countries create false stereotypes and might breed conflict.

Significance Statement In our contemporary globalized world, conflict between citizens and foreigners might hamper efforts to tackle global problems and potentially endanger international relations. Here, we investigate when and how citizens compete with foreigners, and how historical and socio-economic factors shape economic conflict. In an experiment across 51 societies, we found that people systematically failed to anticipate the competitiveness of foreigners. These conflict misperceptions varied substantially across the globe, and were associated with differences in the wealth of nations, quality of institutions, and histories of engaging in international conflict. These results point to important obstacles for international relations.

## Introduction

With the formation of nation states, people across the globe have become divided into citizens and foreigners (1). Due to increases in mobility, cross-country economic activities, and the rise of the internet, citizens increasingly meet and interact with foreigners, both in person and virtually (2–5). These interactions can be peaceful and cooperative (3, 6, 7). Sometimes, however, they are marked by conflict (8–10). Whether grounded in accurate assessments or inaccurate stereotypes, conflict between citizens and foreigners can shape a nation’s policy on, for example, migration and foreign aid (11), and might hamper efforts to solve global problems through coordinated collective action and cooperation (2).

Decades of research have tried to understand social interactions between citizens and foreigners. One approach considers whether and how between-country variation in individual cooperation and honesty associates with historical and socio-economic factors such as ecological stress, economic growth, and the quality of social institutions (2, 12, 13). Another approach investigates trust and cooperation when individuals from different countries interact with each other (7, 14). Interestingly, this line of work shows that individuals often fail to anticipate how cooperative and trustworthy foreigners are (14–16). Misperceptions may be particularly important when individuals compete with and defend against competition from foreigners, and such misperceptions can create and escalate conflict and its waste (17–20). However, as past work almost exclusively focused on trust and cooperation and ignored conflict and competition between citizens and foreigners, we poorly understand when and how citizens compete with foreigners, and how historical and socio-economic factors shape economic competition and conflict.

**Fig. 1.**
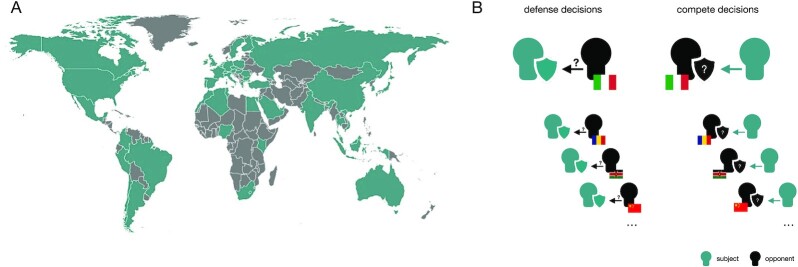
Participating countries and design summary. World map of the citizens’ countries that took part in this study (A). Summary of the design (B). In the contest, participants (green avatar) invested own money in two randomized blocks (see also “Methods” section). In one block, they were asked (using neutral language) how much of their money they want to invest to defend themselves against their opponent’s attempt to take away their money (“defense decisions”). In the other block, participants could invest their money to attempt to take away the money from an opponent (“compete decisions”). In each round, participants faced a different opponent that was extracted from the pool of countries participating in the study and identified by their home-country.

Here, we focus on the determinants of costly competition towards foreigners, and defending against competition from foreigners. A total of 12,863 participants from 51 nations engaged in a cross-country dyadic contest in which they could invest in competition towards their opposing contestant or, reversely, defend against their opponent’s competitive investment (Fig. 1A). Participants were paired with different opponents who were identified by their home-country. They were asked to make several independent conflict decisions without feedback. For each opponent, participants could invest their own money to attempt to take their opponent’s money, as a measure of competition. They also had to decide how much of their money to invest to prevent others from taking their money, as a measure of protective defense (see Fig.   1B and the “Methods” section) (21–23).

On average, participants invested 54% of their endowment into conflict—exceeding rational choice predictions (i.e., 30%; see *Game theoretic analysis* in the “Methods” section) (21). Since competing was costly and conflict investments were nonrecoverable, on average, 59% of the total endowment was wasted in these cross-country competitions. Remarkably, we find that citizens invested systematically more (or less) in defending against foreigners than was warranted by foreigners’ actual competitiveness. This misalignment points to systematic conflict misperceptions across nations (see SI Section 3.1). When we probed the origins of these conflict misperceptions, we observed that, in contrast to widespread opinion, prominent cultural and ecological factors are only weakly related to misaligned investments in competition and defense. Instead, we find that the wealth of nations, the quality of their institutions, and their historical involvement in conflict are robust predictors of conflict misperceptions.

## Results

### Competition vs defense

People invested more resources in defending (*M* = 5.438, SD = 2.512) than in competing (*M* = 5.362, SD = 2.567; mixed-effects regression: *b* = 0.076, *P* < 0.001; Table S1). Younger people invested more resources than older people in both competition and defense (competition: *b* = -0.086, *P* < 0.001; defense: *b* = -0.114, *P* < 0.001; Tables S2 and S3). In line with past research on gender differences and competition (24), women invested less resources in both competition and defense than men (competition: *b* = -0.308, *P* < 0.001; defense: *b* = -0.258, *P* < 0.001; Tables S2 to S4).

### Conflict misalignments

To quantify conflict misalignment across the globe, we defined a measure *m* as the difference between two parameters *α_x_* and *β_x_*, where *α_x_* reflects how much citizens from country *x* invested in competition towards foreigners (i.e., participants from all countries except their own country), and *β_x_* reflects how much foreigners from all countries (except country *x*) invested in defense against the (expected) competition from citizens of country *x*. If individuals have accurate estimates of the investment in competition by a foreigner (*α_x_*), their best response is to invest this amount in defense (*β_x_*) (see the “Methods” section). In this case, investment in competition and defense would be aligned and *α_x_ − β_x_* = 0. Accordingly, any deviation from 0 indicates that participants either expect more competition from foreigners *x* than is actually the case (*β_x_−α_x_* > 0) or that they anticipate less competition from foreigners *x* than is actually the case (*β_x_−α_x_* < 0). At the global level (i.e., averaged across the 51 countries in our sample), the degree and direction of conflict misalignment is reflected by *m_x_* = *β_x_−α_x_*, where *m_x_* > 0 indicates that participants from all countries (except for participants from country *x*) invest on average more than necessary in defense when paired with people from country *x* and *m_x_* < 0 indicates that participants from all countries (except for participants from country *x*) invest on average less than necessary in defense when paired with people from country *x* (Fig. S2).

**Fig. 2.**
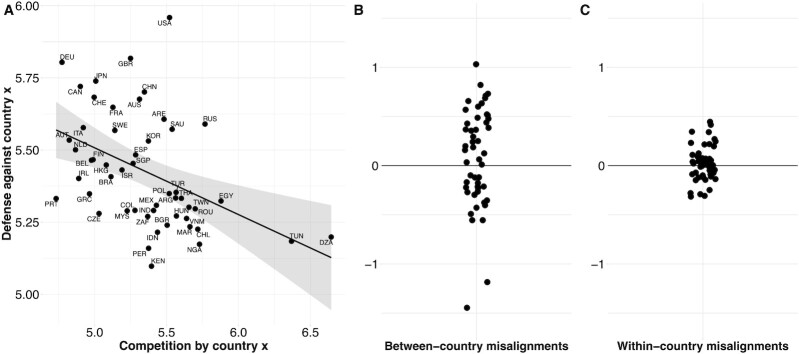
The degree and direction of conflict misalignment across the globe. Scatterplot showing the 51 countries in our sample and its relation between citizens’ investment in defense against foreigners (i.e., defense against country *x*) and actual investment in conflict by people from country *x* (i.e., competition by country *x*). Dots represent country level means and are labelled by country iso code 3 (see Table S51). Gray area represents the 95% CI of the regression line. The negative correlation reveals that conflict investments are systematically misaligned, meaning that participants systematically over- or underestimate the need to protect against the competitiveness of foreigners (A). Jittered scatterplot showing the difference between defense against participants from a foreign country and the actual competition of that country against foreigners (conflict misalignment between countries) (B). Jittered scatterplot showing the difference between defense against and competition towards people from the own country (within-country misalignment) (C). The higher variance in conflict misalignment between countries compared to within countries shows that misperception of conflict is higher in across-country than within-country interactions.

At the global level, we observed a strong negative association between how much foreigners defended against an opponent of a particular country and how competitive the citizens of that country actually were (*r*[49] = -0.442, *P* = 0.001) (Fig. 2A, also see Fig. S7). This means that people around the world were more defensive towards citizens from countries that were actually less competitive, and less defensive towards citizens from countries that were actually more competitive. These findings show that defense towards foreigners is systematically misaligned with their actual competitiveness, pointing to globally shared conflict misperceptions (see SI Section 3.1). Furthermore, between-country conflict misalignments were not only omnipresent but also varied widely in their direction. For some countries, foreigners invested more in defense than necessary, given the actual investment in competition from citizens of these countries (*m_x_* > 0, countries above the 0 line, Fig. 2B). For other countries, foreigners systematically defended too little and misjudged the actual competitiveness of foreigners from country *x* (*m_x_* < 0) (countries below the 0 line, Fig. 2B). Finally, conflict misalignments were significantly larger in interactions between countries than in interactions within countries (Fig. 2C; also see Figs. S9 and S10).

### Cross-societal variation in conflict misalignments

Past research theorized that social interactions around the globe systematically co-vary with environmental and social ecologies (25–27). Variation across environmental and social ecologies could thus be associated with the two sources of conflict misalignments: between-country differences in competition and investments in defense conditional on the nationality of the opponent. We examined this possibility focusing on four prominent sources of socio-ecological variation that have been associated with between-country variation in cooperation and trust in previous research: cultural orientation, ecological stress, historical engagement in international conflict, and socio-economic conditions (see SI Section 1.11 and Fig. S1) (25–27). For our analyses, we retrieved indicators related to these cross-societal factors from past cross-cultural research (3, 14). We estimated the unique contribution of each cross-societal factor by controlling for the others (note that robustness checks excluding two potential outliers seen in the bottom part of Fig. 2B did not change the reported results; see SI Section 1.11.1.1).

Work in cross-cultural psychology and political science indicate that countries with traditional values have more interstate conflicts than countries with more secular values (28–31). Hence, traditional values might relate to how competitive citizens from that country are towards foreigners. Additionally, people might defend more against foreigners living in countries with higher scores on traditional values. We find little evidence for these possibilities. Using a country-level indicator of traditional vs secular values retrieved from the *word value survey* (32), we failed to detect an association between traditional vs secular cultural orientation and competition. We also do not find statistical evidence that participants condition their defense decisions on the traditional vs secular values of the opponent’s country (*P* = 0.765 and 0.823, Tables S5 and S6). Results did not change when considering alternative indicators of cultural orientation (see Tables S27 and S28).

Another possible source of conflict misperceptions pertains to country-level ecological stress. Work in political geography and climate science suggests that regions with higher risk of natural disasters, resource scarcities, and prevalence of infectious diseases are politically less stable, have higher crime rates, and more frequent conflict (33–35). Therefore, country-level ecological stress may correlate with higher investments in competition towards foreigners (36), and people may invest more resources in defending against citizens from foreign countries with higher rather than lower ecological stress. Similar to cultural values, however, we found little evidence for these possibilities. A country’s ecological stress index was neither robustly associated with investment in defense conditional on the opponent’s country (*P* = 0.551, Table S5), nor with investment in competition against foreigners (*P* = 0.726, Table S6). Also, when using indicators of ecological stress from different sources, we do not find statistical evidence that cross-country variance in ecological stress systematically co-varies with defense or competition decisions (see also robustness checks reported in Tables S17 to S28).

**Fig. 3.**
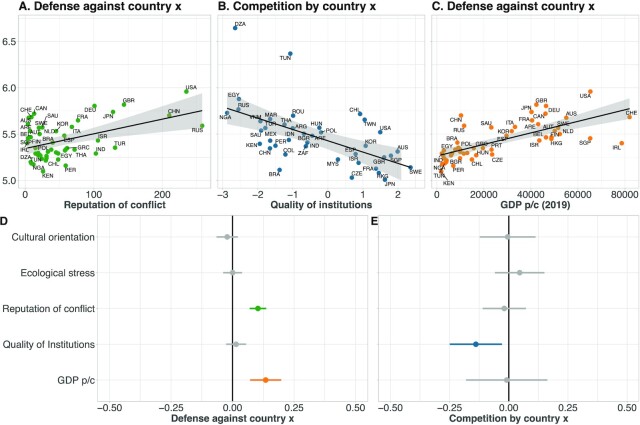
Cross-societal associations and forest plot of regression estimates. Citizens invest more in defense towards people from countries with more frequent historical involvement in international conflict (conflict reputation) (A). Competition by citizens from country *x* towards foreigners is negatively related to the country-level quality of institutions (B). Country-level Gross Domestic Product per capita (GDP; yellow) is positively associated with defense against citizens from country *x* (C). Dots represent country level means. Gray area represents the 95% CI of the regression line. Forest plot of the regression estimates and SEs of a regression model including all country-level cross-societal indicators predicting defense investments against country *x* (D), and competition by citizens from country *x* (E). Points represent coefficient estimates and lines represent 95% CIs. The vertical black line separates significant from nonsignificant coefficients.

A third possible mechanism underlying conflict misperception is a country’s historical engagement in international conflict. When lacking information about foreigners’ behavior, people may be more defensive when facing people residing in countries with a higher rather than lower frequency of being engaged in international conflicts throughout history (37–40). Results are in line with this hypothesis. We retrieved data on militarized interstate disputes spanning the 19th and 20th century (41) and found that people invested more in defense when interacting with foreigners from countries that in the past have been more often involved in international conflicts (*b* = 0.105, *P* < 0.001, Table S5, Fig. 3A). These results were replicated when we used log-transformations of such historical “conflict reputation” and were independent of whether we considered historical conflicts that were in the more recent or more distal past (see SI Section 1.11.2). Strikingly, there was no association between a country’s past engagement in international conflict and its citizens’ investments in competition towards foreigners (*P* = 0.823, Table S6). This shows that conflict misalignment and underlying misperceptions partly arise from a nation’s historical conflict reputation. Citizens’ actual competitiveness toward foreigners around the world was not associated with their country’s historical involvement in international conflict (and therefore also not reflecting decisions of their past political leaders). Differences in historical conflict involvement across countries should therefore not inform defense decisions. Yet, our data provides evidence that foreigners take historical conflict reputations into account when calibrating their level of defense against citizens from these countries.

Finally, we considered a fourth source of societal variation—economic well-being and the quality of institutions as reflected in government effectiveness, rule of law, and democracy within a country. Past work already discussed how social institutions have shaped modern large-scale conflict across societies (25, 33, 35, 42–49). Previous research has also shown that institutional quality and the economic well-being of a country can influence peoples’ cooperativeness and trust (12). Therefore, people living in countries with greater wealth or institutional quality may be less competitive towards foreigners than people from countries with lower economic or institutional conditions (44). Indeed, our quality of institutions measure was negatively associated with competition towards foreigners. People living in countries with stronger democracy, greater rule of law, and greater government effectiveness invested less in competition towards foreigners than people residing in countries with relatively lower quality of institutions (*b* = -0.130, *P* = 0.032, Table S6, Fig. 3B). Interestingly, while GDP did not systematically predict actual competitiveness of citizens (*P* = 0.782, Table S6), people invested more in defense against foreigners living in richer countries compared to foreigners living in poorer countries (*b* = 0.119, Table S5, *P* = 0.001, Fig. 3C). This finding was robust across different GDP levels, across a diverse set of indicators related to wealth, and across a wide set of indicators related to the quality of institutions, including different aspects of democracy (Tables S29 to S38).

We replicated these cross-societal results with different analytic strategies, such as models that allow to take into account individual and country-level variation and that control for relevant individual socio-demographics such as age, gender, and education (see SI Section 1.5). These additional analyses provide consistent evidence on the pervasiveness of conflict misperceptions, and their potential cross-societal origins. Moreover, our results do not change when we use additional cross-societal indicators that were retrieved from different sources, suggesting that our results are robust to different operationalizations of these constructs (see SI Section 1.11). Finally, results replicate in a subset of only western countries and only nonwestern countries, thus excluding a “west against the rest” interpretation (1) (see SI Tables S12a and b, Figs. S5 and S6). A summary of the cross-societal regression effects is shown in Fig. 3D and E.

### Bilateral distances

Thus far, we analyzed how country-level characteristics of foreigners influence defense decisions against those foreigners, revealing what country-level information participants seem to integrate when making their defense decision against a particular opponent. By looking at the degree to which country-level characteristics actually relate to differences in competitiveness across countries, we were able to reveal potential sources of why competitiveness of citizens from country *x* is frequently misaligned with defense decisions against people from country *x* (i.e., Fig. 2). For example, participants invested more in defense when their opponent was from a country involved in more historical disputes, while variation in historical conflict did not significantly explain variation in competitiveness of citizens across countries.

While these analyses can shed light on the general pattern of conflict misalignment, we observe across all countries included in this study, they do not allow us to explain bilateral misalignment in competitiveness and defense between two countries. We therefore investigated whether bilateral (i) socio-psychological, (ii) genetic, or (iii) geographical distances between countries were associated with bilateral conflict misalignments. As additional measures of bilateral distances, we also considered bilateral trade and migration flow (see SI Section 1.13 for details about these bilateral measures). Across the world, socio-psychological distance between countries (an index of difference between two countries in terms of norms, values, behaviors, and attitudes) was the only factor associated with larger absolute bilateral conflict misalignments (*b* = 1.933, *P* < 0.001; see Table S50). This illustrates that socio-psychological distance between two countries can exacerbate wrong stereotypes and beliefs in conflict. Together with the cross-societal analyses on investment in competition and defense, it follows that these bilateral distances partially originate from differences in institutions, wealth, and historical reputation of conflict.

## Discussion

Our data show that costly investments in competition and defense between citizens from different countries are prevalent and often marked by misperceptions. While competition towards foreigners is negatively associated with social institutions, like rule of law or democratic governance, citizens are actually more cautious and invest more in defense when facing people from more prosperous countries or countries with more frequent historical engagements in international conflict. Yet, neither of the latter indicators reliably predicted how much the citizens of these countries actually invested in competition. In other words, citizens across the globe systematically over- or underestimate foreigners’ competitiveness based on shared but uninformative country-level reputations and wrong stereotypes. As such, participants fall for an ecological fallacy. Their defense decisions against opponents of a particular country are systematically influenced by country-level characteristics that are not systematically related to the actual behavior of its citizens. Contrary to popular viewpoints (1, 29), defense and competition were weakly associated with a country’s cultural traditional values or its ecological stress when controlling for other factors. Rather, conflict misperceptions appear to originate in wrong beliefs about the competitiveness of people living in wealthy countries and countries with strong social institutions.

Our analyses were guided by past theory that hypothesized that socio-ecological differences are associated with between-country differences in conflict. Results are robust across different operationalizations of cross-cultural constructs and replicated using analytic strategies that consider within country and individual variation. Nonetheless, findings and conclusions should be considered in light of a few potential limitations. First, one might argue that economic contests do not provide an ecologically valid measure of the willingness to engage in conflict in real life, due to their abstract nature and small (financial) stakes. To address this potential issue, we cross-validated our findings with a self-report measure of conflict collected in an independent cross-cultural dataset: the world values survey (32). We observed that both competing against and investing in defense were positively associated with the degree to which people respond “yes” to the question “Would you be willing to fight for your own country?” (competition towards foreigners: *r* = 0.478, *P* < 0.001, Fig. S3; defense against foreigners: *r* = 0.516, *P* < 0.001, Fig. S4, see SI Section 1.8). This suggests that our cross-cultural dataset can successfully detect between-country differences in related conflict measures that more directly probe conflict intentions. Second, we used country-level indicators to understand how variation across socio-ecologies can contribute to conflict misperceptions. Although country-level indicators may not perfectly reflect individual-level characteristics, our main results on misalignment do not rest on inferences from country-level to subject-level data. Instead, we analyzed how decisions in the experiment are influenced by prominent macro-level contextual differences such as wealth and the quality of institutions (that people may use as a heuristic or stereotype when interacting with foreigners from these societies). Lastly, and relatedly, while the country-level analyses can indicate potential factors that contribute to the formation of conflict misalignments, these analyses do not allow us to make causal conclusions. Future research is needed to understand the relation between these cross-cultural factors and their causal effects on actual conflict and conflict misperception.

Whereas we identified how some cross-societal variations are, and others are not associated with competing and defending against foreigners, there may be additional sources of relevant cross-societal variations, such as historical roots of institutional differences or the role of current international affairs. We surmise that such additional factors would be particularly relevant for explaining between-country differences in competition, as the model with institutions explained 46% of variance (compared to 73% of explained variance in the model predicting defense against country *x* based on wealth and historical reputation, SI Section 1.3). This indeed emerged from exploratory analyses that included kin-based institutions—defined by the prevalence of cousin-marriage preferences, polygamy, co-residence of extended families, clan organization, and community endogamy (50)—and the share of GDP spent on military. We find that both kinship institutions and GDP share associate with between-country differences in competition, but not between-country differences in defense (see Tables S39 and S46, Fig. S8). These findings provide further support for the conclusion that variation across institutions and wealth might be associated with conflict misperceptions around the world.

Conflict misperceptions can create and escalate conflict and polarize intergroup relations (17, 18, 20). Incorrect stereotypes may fuel nationalistic sentiments and threaten relations between citizens and foreigners around the world. Reducing economic inequalities between countries and enhancing the quality of institutions provides, however, a promising avenue to reduce conflict misperceptions and costly competition between citizens across the globe.

## Methods

The research and procedure (including the informed consent obtained by all participants) were approved by the Psychology Research Ethics Committee of Leiden University, application number: 2020-02-03-A. Romano-V1-2068.

### Participants

We collected data from 12,863 participants across 51 nations (Algeria, Argentina, Australia, Austria, Belgium, Brazil, Bulgaria, Canada, Chile, China, Colombia, Czech Republic, Egypt, Finland, France, Germany, Greece, Hong Kong, Hungary, India, Indonesia, Ireland, Israel, Italy, Japan, Kenya, Korea, Malaysia, Mexico, Morocco, Netherlands, Nigeria, Peru, Poland, Portugal, Romania, Russian Federation, Saudi Arabia, Singapore, South Africa, Spain, Sweden, Switzerland, Taiwan, Thailand, Tunisia, Turkey, United Arab Emirates, United Kingdom, United States, and Vietnam). Participants were recruited through the Toluna Panel, including members of its third-party panel providers. Participants were stratified by age and gender. Our goal was to recruit 12,750 participants (∼250 per society). A sensitivity power analysis showed that 250 people can detect a small effect size of *d* = 0.25 with 80% power (between-country difference of competition or defensiveness).

### Preregistration

The experimental setup, measures, and analytic strategy were preregistered at https://osf.io/nf7ks/?view_only = 1562f490520f4b5b90320185b2bbd445. We note that the current focus is on preregistered hypotheses of section 1 and 4 of the preregistration (and the unexpected, not preregistered result on conflict misalignments), and that the preregistration includes hypotheses pertaining to distinctly different research questions that we do not cover here. Accordingly, we do not report results from some of the measures that are mentioned in the preregistration and from a few decisions collected in the attacker–defender game (i.e., unidentified stranger). The additional measures are: suffering from the corona pandemic, national identity, generosity, political ideology, interdependence, and other social preferences (e.g., inequality aversion).

### Procedure and general design

The design consisted of two within-subject treatments related to the role of the participant (Participant’s role: “attacker” vs “defender,” see below) and the opponent that the participant is interacting with (identified by the opponent’s nationality, randomly selected from the pool of 51 nations participating in the study). The experiment was administered through an online survey. We wrote an English version of the survey and asked experts and professional translators to translate the survey. The procedure of the experiment was the same across all countries. After giving their informed consent, participants were asked to make 52 independent decisions, facing different opponents across the world. No feedback about others’ decisions was provided. Participants could not face multiple opponents from the same foreign country. Thereafter, participants also responded to several additional questionnaires, unrelated to this project, and asked to give information about their gender, age, and education. We note that throughout the instructions we used neutral language and avoided terms like competition, defense, opponent, or conflict (51).

### Investing in competition and defense

We assessed competition towards and defense against foreigners in the context of economic contests. The contest models conflict and competition between an “Attacker” and a “Defender” (attacker–defender contest; AD-C) (22, 23). Participants are given an initial endowment of 10 Monetary Units (MU) and are assigned a role (attacker or defender; in the instructions labeled as Person A and Person B, respectively). Both attacker and defender decide how many of the 10 MU they want to invest into a challenge pool (investment = *i_x_* 0 ≤ *i_x_* ≤ 10) or keep for themselves. If the investment of the attacker is higher than the investment made by the defender (*i*_att_ > *i*_def_), the attacker’s final earnings (π_att_) are the remaining endowment not invested into conflict plus the endowment the defender kept for themselves: π_att_ = (10 – *i*_att_) + (10 – *i*_def_). In other words, the attacker takes the remaining resources of the defender and the defender ends up with nothing: π_def_ = 0. However, if the investment to the challenge pool of the defender is equal or greater than the investment of the attacker (*i*_att_ ≤ *i*_def_), both attacker and defender simply end up with the MU they did not invest in the challenge pool in the first place: π_att_ = *e*_att_*− i*_att_; π_def_ = *e*_def_*− i*_def_. In other words, the defender successfully defends their remaining resources from the attack of the opponent. Hence, participants, depending on their role, can attempt to take away resources from the other person (degree of competitiveness) or defend against such attempts.

### Game-theoretic analysis

Assuming rational selfish play and risk neutrality, the AD-C has a unique Nash equilibrium in mixed strategies, such that players should randomize their investment (up to a certain threshold) to maximize their payoff. This means that in the AD-C there is not a clearly advantageous action. The benefits of investing in conflict depend on the investments made by the opponent and vice versa (21, 23). The best-response of defenders is to match attackers’ investments. On the other hand, for attackers, the best response would be to invest exactly either one unit more than the defenders or to not invest in attack at all, depending on whether the remaining capital not invested by attackers and defenders is large enough to make an attack investment worthwhile.

To characterize the strategies played in the mixed Nash equilibrium with an equal starting endowment of *e* = 10 for attackers and defenders, we denote *P*(*X*) as the probability of investing *X* by attackers, and *P*(*Y*) the probability of investing *Y* by defenders. A strategy assigns a probability value for each possible action (i.e., investment). In equilibrium attackers should choose: *P*(*X* = 1) = 2/45, *P*(*X*) = *P*(*X*–1)*[(12–*X*)/(10–*X*)] for 2 ≤ *X* ≤ 6, *P*(*X* = 0) = 1–[*P*(*X* = 1) +. . .*P*(*X* = 6)] = 0.4, and *P*(*X*) = 0 for *X* ≥ 7, i.e.; *P*(0) = 0.4, *P*(1) = 0.0$\bar 4$, *P*(2) = 0.0$\bar 5$, *P*(3) ≈ 0.0714, *P*(4) ≈ 0.0952, *P*(5) = 0.1$\bar 3$, *P*(6) = 0.20, *P*(7) = 0, *P*(8) = 0, *P*(9) = 0, *P*(10) = 0. Defenders should choose: *P*(*Y*) = 1/(10-*Y*) for 0 ≤ *Y* ≤ 5, *P*(*Y* = 6) = 1–[*P*(*Y* = 0) +. . .+ *P*(*Y* = 5)] = 0.15, and *P*(*Y*) = 0 for *Y* ≥ 7, i.e.; *P*(0) = 0.1, *P*(1) = 0.1$\bar 1$, *P*(2) = 0.125, *P*(3) = 0.1428, *P*(4) = 0.1$\bar 6$, *P*(5) = 0.2, *P*(6) = 0.15, *P*(7) = 0, *P*(8) = 0, *P*(9) = 0, *P*(10) = 10. For further details about equilibria in attacker–defender game, see ref. (52). Figure S11 provides a graphical representation of the strategy profiles in equilibrium. If we calculate the expected investments from these probabilities, attackers should invest *i*_att_ = 2.62, while defenders should invest *i*_def_ = 3.38 on average in equilibrium. We used these numbers as benchmarks to characterize over-investments.

### Treatments

For each decision, participants were assigned to interact with a person that was randomly selected from the pool of 51 nations included in the study. Before making their decision, they were informed about their partner’s nationality. For the present study, we had 52 decisions divided in two (randomized) blocks of 26 decisions, varying whether the decision was made in the role of attacker (attacker treatment), or in the role of defender (defender treatment). For each block, one decision involved interacting with a person of the same nationality, while the other 25 decisions involved persons with a different nationality. Each nationality was randomly extracted once, such that participants could only make one decision as attacker and one decision as defender with a person of a specific nationality. The nationalities of the persons encountered in the second block matched those presented in the first block. Overall, the frequency of extracting the opponents’ nationalities was balanced across participants, such that we have an equal number of nation–nation pairs across the sample. We collected a total of 656,274 decisions among 12,863 participants from 51 countries.

### Incentives

To make decisions comparable across nations in terms of earnings, each MU was worth 1 minute of the average hourly wage in their country. Therefore, each participant started with an amount corresponding to 10 minutes wage in their nation. Information of wage in each nation were retrieved from https://tradingeconomics.com/country-list/wages. Participants were paid for one role and one of their decisions in that role, randomly chosen. Participants were told that they would make decisions in both roles (attacker and defender; labeled as Person A and B in the experiment) and that, at the end of the experiment, we would randomly match each participant with another participant from the respective country and that their decisions would both affect their own earnings as well as the earnings of their randomly selected other party.

### Cross-country indicators

To explain cross-country variation in competition (investments as attackers) and defense as a function of own nationality and the nationality of the other person, we considered cross-country indicators related to differences in cultural values, ecological stress, a nation state historical involvement in conflict, as well as quality of institutions and economic well-being. Below, we report a description of the indicators used in the main analyses. In the SI (see Section 1.11), we report analyses with other operationalizations of the same constructs.

### Cultural orientation (traditionalism vs secularism)

In line with previous cross-societal research (e.g. 53), we specified country-level cultural values by retrieving the traditional vs secular dimension measure from the longitudinal world values survey 1981 to 2020 (32). Traditional values emphasize the importance of religion, parent–child ties, deference to authority, and traditional family values. These societies have high levels of national pride and a nationalistic outlook.

### Ecological stress

The ecological stress indicator is a composite score of two intercorrelated cross-societal indicators (*r* = 0.37; see also SI Section 1.11): historical prevalence of infectious disease (54), and vulnerability to natural disasters. Historical prevalence of infectious diseases (e.g., leishmanias, schistosomes, and trypanosomes) was extracted from Murray and Schaller (54). Natural disaster vulnerability is an indicator of the frequency, severity, and number of deaths due to natural disasters (55).

### Reputation for historical involvement in international conflict

We retrieved this indicator from the Correlates of War database (41). For each country, we calculated the total number of documented militarized disputes from 1816 to 2007.

### Quality of institutions

To operationalize quality of institutions, we extracted three dimensions of governance: rule of law, government effectiveness, and democracy (see Table S16). Then, we used a principal component analyses to extract a unique measure of quality of institutions. Rule of law represents perceptions of the extent to which people have confidence in and abide by the rules of society. Government effectiveness captures perceptions of the quality of public services, the quality of the civil service, and the degree of its independence from political pressures, the quality of policy formulation and implementation, and the credibility of the government’s commitment to such policies. Democracy is a composite index of the degree to which a country promotes pluralism, functioning of government, political participation, political culture, and civil liberty.

### Economic well-being

Economic well-being was operationalized as the gross domestic product per capita from 2019 (retrieved from the World Bank; http://data.worldbank.org/indicator/).

### Analytic strategy

For the main treatment effect (competition towards vs defense against competition), we used mixed-effects models in which participants (level 2) and nations (level 3) are two random factors. These models consider random intercepts for participants nested in nations. We analyzed data with *R* (lme4 package) and used random intercept (56). Individual differences variables (e.g., age and gender) were level-2 controls.

Regarding the cross-societal analyses, we performed simple regressions of country-level indicators predicting country-level investment in defense and in competition for the main analyses. We used multiple imputations methods for indicators where we had missing cases (package *mice*). Additional analytic strategies used for robustness checks are detailed in the Supplementary Information.

## Supplementary Material

pgac267_Supplemental_File

## Data Availability

Data (aggregated at the country-level) and R codes are available in the Open Science Framework at https://osf.io/kuc45/.
