# Supplementary material for: Conflict misperceptions between citizens and foreigners across the globe
Source: PNAS Nexus. 2022 Nov 22;1(5):pgac267. doi: 10.1093/pnasnexus/pgac267 (PMC9802172; doi:10.1093/pnasnexus/pgac267)
Supplement: pgac267_Supplemental_File [file pgac267_supplemental_file.docx]

Supplementary Materials for

**Conflict Misperceptions between Citizens and Foreigners across the Globe**

Angelo Romano, Jörg Gross, Carsten K.W. De Dreu

Correspondence to: a.romano@fsw.leidenuniv.nl

1. **Supporting analyses**

1.1. Interindividual differences

1.2. Competition and Defense

1.3. Cross-societal models

1.3.1. Defense against country *x*

1.3.2. Competition by country *x*

1.4. Multi-collinearity

1.5. Cross-societal mixed effects model

1.6. Misperceptions and cross-societal indicators

1.7. Independent t-tests competition vs. defense

1.8. Cross-validation with world value survey

1.9. Cultural clusters

1.9.1. West against the rest

1.10. Cross-societal models without imputation

1.11. Cross-societal models with additional indicators

1.11.1. PCA institutions, ecology, and wealth

1.11.1.1. Potential outliers: Algeria and Tunisia

1.11.2. Log reputation of conflict, recent and distant reputation of conflict

1.11.3. Cultural clusters

1.11.4. Democracy

1.11.5. NATO membership, share of military budget from GDP, and recent involvement in international conflict

1.11.6. Other potential cross-societal factors

1.12. Conditional conflict across different GDP levels

1.13. Bilateral distances

1.14. Misalignment within vs. between countries

1. **Descriptives**
2. **Game theory**

3.1. Conflict misalignments and conflict misperceptions

**References**

1. **Supporting analyses**

In this section, we provide a full report of the statistical models we fitted to our data. First, we present the models with the main treatment and controls only: i.e., investment to defend own resources vs. investment to take resource from others (Table S1).

- 1. **Interindividual differences**

The first model consists of the main treatment predicting investment in conflict: Defense vs. Competition (Defense = 1, Competition = 0), Age (continuous variable in years), Gender 1 (1 = Women, 0 = Men), Gender 2 (1 = Other, 0 = Men), Education (ordinal variable where 1 = elementary school, 2 = middle school, 3 = high school, 4 = some college, 5 = bachelor degree, 6 = graduate school or higher). In this model, participant ID and countries were random intercepts. In line with game-theoretic predictions and previous laboratory experiments (1, 2), results show that people invest more in defense than in competition. As a result, defending was also more successful than competing (56% vs 44%). We also find that men, compared to women, invest more in conflict (investment in defense and competition combined), that age is negatively associated with conflict and that more educated people invest more in conflict, compared to less educated people. Potential explanations for these individual differences, including differences in beliefs and risk preferences, can be probed in future research.

**Table S1.** Mixed-effect model of defense (vs. competition) predicting conflict.

| **Conflict** | ***b*** | ***SE*** | ***t*** | ***p*** |
| --- | --- | --- | --- | --- |
| Defense (vs. Competition) | 0.076 | 0.004 | 17.317 | <0.001 |
| Age | -0.100 | 0.017 | -5.976 | <0.001 |
| Gender 1 (Women = 1) | -0.282 | 0.032 | -8.727 | <0.001 |
| Gender 2 (Other = 1) | -0.943 | 0.370 | -2.552 | 0.011 |
| Education | 0.083 | 0.015 | 5.470 | <0.001 |

*Notes.* Tests are two-sided. *N*_observations_ = 655,964; *N*_subjects_ = 12,857, *N*_countries_= 51.

25 people responded “Other” in the question about gender.

- 1. **Competition and defense**

In Table S2 to S4, we report a model with demographic variables (gender, age, and education) predicting competition (Table S2), defense (Table S3), and the difference between defense and competition (Table S4), separately. In these datasets, each row represents one subject. Therefore, these models have only country as a random intercept.

**Table S2.** Mixed-effect model of demographic variables predicting competition.

| **Competition** | ***b*** | ***SE*** | ***t*** | ***p*** |
| --- | --- | --- | --- | --- |
| Gender (Woman = 1, Man = 0) | -0.308 | 0.036 | -8.652 | <0.001 |
| Gender (Other = 1, Man = 0) | -1.009 | 0.407 | -2.480 | 0.013 |
| Age (years) | -0.086 | 0.018 | -4.720 | <0.001 |
| Education | 0.082 | 0.017 | 4.942 | <0.001 |

*Notes.* The variable age was standardized. Tests are two-sided. *N*_observations_ = 12,857, *N*_countries_= 51

**Table S3.** Mixed-effect model of demographic variables predicting defense.

| **Defense** | ***b*** | ***SE*** | ***t*** | ***p*** |
| --- | --- | --- | --- | --- |
| Gender (Woman = 1, Man = 0) | -0.258 | 0.035 | -7.366 | <0.001 |
| Gender (Other = 1, Man = 0) | -0.883 | 0.400 | -2.205 | 0.027 |
| Age (years) | -0.114 | 0.018 | -6.338 | <0.001 |
| Education | 0.085 | 0.016 | 5.169 | <0.001 |

*Notes.* The variable age was standardized. Tests are two-sided. *N*_observations_ = 12,857, *N*_countries_= 51

**Table S4.** Mixed-effect model of demographic variables predicting the difference between defense and competition.

| **Defense - Competition** | ***b*** | ***SE*** | ***t*** | ***p*** |
| --- | --- | --- | --- | --- |
| Gender (Woman = 1, Man = 0) | 0.055 | 0.028 | 1.957 | 0.050 |
| Gender (Other = 1, Man = 0) | 0.134 | 0.324 | 0.415 | 0.678 |
| Age (years) | -0.024 | 0.014 | -1.659 | 0.097 |
| Education | -0.001 | 0.013 | -0.031 | 0.975 |

*Notes.* The variable age was standardized. Tests are two-sided. *N*_observations_ = 12,857, *N*_countries_ = 51

- 1. **Cross-societal models**

In this section, we present simple regression models using country-level indicators predicting defensiveness against citizens from other countries and actual competitiveness of citizens from different countries. Actual competitiveness is measured as the mean-level investments within a country in attacking others (independent of the partner’s nationality). Defensiveness against citizens from other countries is the average investment in defense when facing an opponent from a particular country. Our cross-societal indicators were gross domestic product per capita (GDP), institutional quality, reputation of conflict, traditionalism (vs. secularism), and ecological stress. Since it was not possible to retrieve a value for all the 51 countries involved in the study for some indicators, we used the multiple imputation method as implemented in the *mice* package in R. The results of these models are the average estimates of five regressions across five imputed datasets using the function *pool* within *mice* (3).

**1.3.1. Defense against country *x***

Table S5 shows the results of the five cross-societal indicators predicting defense against foreigners from other countries. Results show that across all indicators, gross domestic product per capita and reputation of conflict (i.e., a country’s historical engagement in international conflict) are the only significant predictors. People invested more in defense against partners belonging to countries with greater GDP (vs. lower GDP) and with a reputation of being historically more involved in conflicts (*R*^2^ = 0.73).

**Table S5.** Regression model of gross domestic product, institutional quality, reputation of conflict, traditionalism, and ecological stress predicting defense against country *x*.

| **Defense against country *x*** | ***b*** | ***SE*** | ***t*** | ***p*** |
| --- | --- | --- | --- | --- |
| Gross domestic product | 0.119 | 0.033 | 3.612 | 0.001 |
| Institutional quality | 0.016 | 0.021 | 0.735 | 0.467 |
| Reputation of conflict | 0.105 | 0.017 | 6.196 | <0.001 |
| Traditionalism | -0.008 | 0.026 | -0.305 | 0.765 |
| Ecological stress | -0.012 | 0.020 | -0.604 | 0.551 |

*Notes.* Tests are two-sided. *N*_observations_ = 51.

**1.3.2. Competition by country *x***

Table S6 shows the results of the five cross-societal indicators predicting competition against foreigners across societies. Results show that, across all indicators, institutional quality is the only significant predictor for investments in competition against foreigners. These results suggest that, when looking at the overall competition level across societies, people living in societies with greater institutional quality (greater democracy, rule of law, government effectiveness) invest less of their resources to attempt to take from foreigners than people living in societies with lower institutional quality (*R*^2^ = 0.46).

**Table S6.** Regression model of gross domestic product, institutional quality, reputation of conflict, traditionalism, and ecological stress predicting actual competitiveness.

| **Competition by country *x*** | ***b*** | ***SE*** | ***t*** | ***p*** |
| --- | --- | --- | --- | --- |
| Gross domestic product | -0.028 | 0.101 | -0.280 | 0.782 |
| Institutional quality | -0.130 | 0.058 | -2.231 | 0.032 |
| Reputation of conflict | -0.011 | 0.047 | -0.226 | 0.823 |
| Traditionalism | -0.014 | 0.060 | -0.225 | 0.823 |
| Ecological stress | 0.027 | 0.075 | 0.364 | 0.726 |

*Notes.* Tests are two-sided. *N*_observations_= 51.

- 1. **Multi-collinearity**

One potential concern of the results presented in Table S5 to S6 could be the presence of high correlations among cross-societal indicators (see Figure S1). High correlations between indicators may increase multi-collinearity and influence the stability and interpretation of the regression coefficients. To check for multicollinearity problems, we used the package *car* in R on a single imputed dataset (using the package *missForest,*(4)) with the five main cross-societal indicators predicting defense and competition and computed the Variance Inflation Factor (VIF, Table S7). A cut-off of 10 is usually set to delineate whether there are multi-collinearity issues within a model, with a stricter standard using a cut-off of 5 (5). Our results show that our model satisfied both the standard and the more conservative criterion.


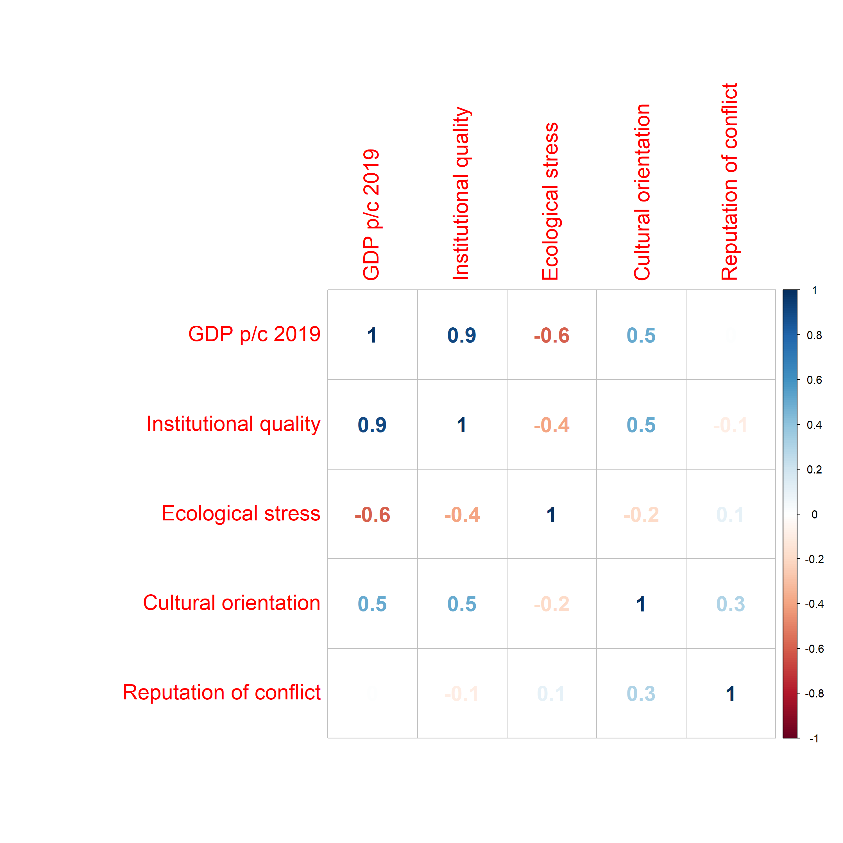


Fig. S1. **Simple correlations among cross-societal indicators.** Very low correlations are not shown (e.g., reputation of conflict and GDP, *r* = -0.04).

**Table S7.** Results from the multi-collinearity check.

| **Indicator** | ***VIF*** |
| --- | --- |
| GDP p/c 2019 | 4.22 |
| Institutional quality | 4.28 |
| Reputation of conflict | 1.15 |
| Traditional vs. secular | 1.53 |
| Ecological stress | 1.48 |

- 1. **Cross-societal mixed-effects model**

Another potential concern with the findings shown in Table S5 and Table S6 is that they do not control for several interindividual and cross-societal variables, and that they are based on a relative low number of observations (51 countries). To address these concerns, this section presents a cross-validation of the findings shown in section 1.3 using a different statistical approach. Instead of aggregating the data to the country-level, we fitted mixed effects regression models using country-level indicators predicting investment in the AD-C. In this model, we include cross-societal predictors (GDP, quality of institution, ecological stress, reputation of conflict, traditional vs. secular values) as fixed effects predicting individual-level investment (both as attacker and as defender). In our individual-level dataset, we integrated each cross-societal indicator with the country of the participant making the investment decision (the investor). We also integrated each cross-societal indicator with the country of the partner in the specific trial (the opponent), thus giving us a dataset that includes country-level indicators of decision-makers as well as opponents. The multi-level structure of the model can be described by the following equation (*Y_ijk_* is either investment in competition or defense from competition). Investment ranges from 0 to 10:

Level 1: *Y_ijkl_* = β_0_*_jkl_* + *e_ijkl_*;

Level 2: β_0_*_jkl_ =* γ_00_*_kl_* + γ_0(1.._*_m_*_)_*_kl_*(DEMOGRAPHICS*_m_*)*_jkl_* + θ_00_*_kl_* + *f*_0_*_jkl_*;

Level 3: γ_00_*_kl_ =* δ_000_ + δ_00(1.._*_n_*_)_(INDICATOR*_n_*)*_kl_* + *g_0kl_*;

θ_000_*_l_ =* η_0000_ + η_00(1.._*_n_*_)_(INDICATOR*_n_*)*_kl_* + *h*_0_*_l_*

In this model, subjects (β, level 2), country of the participant (γ, level 3) and the country of their opponent (θ, level 3) are random intercepts.

With such multi-level model specification, we ran three mixed-effect regressions. In the first model, we included five cross-societal indicators related to the country of the opponent (fixed-effects, level 3). In the second model, we also included the five cross-societal indicators related to the country of the participant (fixed-effects, level 3). Finally, we ran a model including level 2 demographics such as age, gender, and education. We ran such models for both competition and defense from competition. This allowed us to test whether the results presented in section 1.3 hold when controlling for the country of the investor and the country of the opponent. Moreover, we can also control for sociodemographic variables, such as age, gender, and education.

To replicate the results presented in section 1.3, we should observe that the institutional quality of the investor (institutional quality – investor) is the only indicator that significantly predicts investment in competition (towards foreigners). By contrast, we should observe GDP and reputation of conflict to be the only significant predictors of investment in defense (against foreigners). Both results hold in these models. We ran three regressions where we stepwise added different variables. Overall, we consistently found that people invest more in conflict (both competition and defense from competition) with opponents from richer countries and opponents from countries with a historical reputation of being involved in conflict. Participants from societies that score high on institutional quality invested less in conflict compared to participants from countries that on average score lower on the quality of institution indicator. All other cross-societal indicators related to the investors’ decisions are not significant.

**Table S8.** Mixed effect models with cross-societal indicators of investors and opponents predicting competition against foreigners.

| **Competition** | **(1)** | **(2)** | **(3)** |
| --- | --- | --- | --- |
| Gross domestic product – opponent | 0.116(0.03)*** | 0.116(0.03)*** | 0.116(0.03)*** |
| Institutional quality – opponent | 0.025(0.019) | 0.025(0.019) | 0.025(0.019) |
| Reputation of conflict – opponent | 0.105(0.016)*** | 0.105(0.016)*** | 0.105(0.016)*** |
| Traditionalism – opponent | -0.001(0.021) | -0.001(0.021) | -0.001(0.021) |
| Ecological stress – opponent | -0.018(0.018) | -0.018(0.018) | -0.018(0.018) |
| Gross domestic product – investor |  | -0.022(0.088) | -0.044(0.086) |
| Institutional quality – investor |  | -0.134(0.057) | -0.112(0.056)^†^ |
| Reputation of conflict – investor |  | -0.009(0.046) | -0.01(0.045) |
| Traditionalism – investor |  | -0.015(0.061) | 0.002(0.059) |
| Ecological stress – investor |  | 0.023(0.053) | 0.002(0.051) |
| Age (in years) |  |  | -0.08(0.018)*** |
| Women |  |  | -0.309(0.036)*** |
| Other |  |  | -1.02(0.409)* |
| Education |  |  | 0.079(0.017)*** |
| *N_observations_* | 315,274 | 315,274 | 315,125 |
| *N_subjects_* | 12,863 | 12,863 | 12,857 |

*Notes.* Tests are two-sided.^***^*p* < 0.001; ^†^*p* = 0.051

**Table S9.** Mixed effect models with cross-societal indicators of investors and opponents predicting defense from foreigners’ competition.

| **Defense from competition** | **(1)** | **(2)** | **(3)** |
| --- | --- | --- | --- |
| Gross domestic product – opponent | 0.123(0.029) *** | 0.123(0.029)*** | 0.123(0.029)*** |
| Institutional quality – opponent | 0.011(0.019) | 0.011(0.019) | 0.011(0.019) |
| Reputation of conflict – opponent | 0.104(0.015)*** | 0.104(0.015)*** | 0.104(0.015)*** |
| Traditionalism – opponent | -0.007(0.02) | -0.007(0.02) | -0.007(0.02) |
| Ecological stress – opponent | -0.014(0.017) | -0.014(0.017) | -0.014(0.018) |
| Gross domestic product – investor |  | 0.033(0.089) | 0.007(0.087) |
| Institutional quality – investor |  | -0.15(0.058)* | -0.123(0.057)* |
| Reputation of conflict – investor |  | -0.018(0.046) | -0.016(0.046) |
| Traditionalism – investor |  | 0.01(0.061) | 0.028(0.06) |
| Ecological stress – investor |  | 0.037(0.053) | 0.014(0.052) |
| Age (in years) |  |  | -0.108(0.018)*** |
| Women |  |  | -0.258(0.035)*** |
| Other |  |  | -0.886(0.402)* |
| Education |  |  | 0.081(0.017)*** |
| *N_observations_* | 315,274 | 315,274 | 315,125 |
| *N_subjects_* | 12,863 | 12,863 | 12,857 |

*Notes.* Tests are two-sided.^***^*p* < 0.001; **p* < 0.05

**1.6. Misperceptions and cross-societal indicators**

As an additional analysis, we also ran the same cross-societal model presented in the Table S5 and S6 with a different outcome variable. We computed the difference between defensiveness and actual competitiveness across countries (*m_x_*). Such results can directly reveal which cross-societal factors explain the difference between defense against a particular country and its actual competitiveness (i.e., the direction of conflict misalignments). We find that institutional quality and reputation of conflict are the only significant predictors. People living in societies characterized by greater (vs. lower) quality of institutions tend to be perceived as more competitive (i.e., defended against more) than they actually are. Moreover, people living in countries with a reputation for being historically involved in conflict in the past are perceived as more competitive than they actually are, compared to people from countries without such a reputation.

**Table S10.** Regression model of gross domestic product, institutional quality, reputation of conflict, traditionalism, and ecological stress predicting misperceptions.

| **Conflict Misalignment** | ***b*** | ***SE*** | ***t*** | ***p*** |
| --- | --- | --- | --- | --- |
| Gross domestic product | 0.128 | 0.091 | 1.402 | 0.168 |
| Institutional quality | 0.143 | 0.058 | 2.464 | 0.018 |
| Reputation of conflict | 0.002 | 0.001 | 2.322 | 0.025 |
| Traditionalism | 0.026 | 0.064 | 0.406 | 0.686 |
| Ecological stress | -0.112 | 0.098 | -1.146 | 0.258 |

*Notes.* All tests are two sided.

**1.7. Independent t-tests; competition vs. defense**

In this section, we show the degree of conflict misalignments around the world by country (Figure S2) and the independent t-tests of conflict misperceptions within each country (Table S11). Table S11 shows the sample sizes, t-, and p-values of independent t-tests for differences between participants’ competition within a country (competition against foreigners) and participants’ investments to defend against that particular country (defense against foreigners). For each country, competition data was extracted from each individual’s decision within a country to invest in attack towards all their foreign partners while defense data represents the investments made by all individuals across other countries to defend against that particular country (excluding within-country decisions). Significant effects associated with positive t-values represent countries that were more competitive than expected, while significant effects associated with negative t-values represent countries that were less competitive than expected. The reported p-values were not corrected for multiple hypotheses testing. However, even if we apply a very conservative correction (Bonferroni 0.05/51 = 0.00098), we still observe a significant difference in 43 out of 51 countries (countries that would not be significant after Bonferroni correction are India, Korea, Czech Republic, Malaysia, Mexico, Saudi Arabia, South Africa, United Arab Emirates). In sum, these findings show that conflict misperceptions between countries are wide-spread.


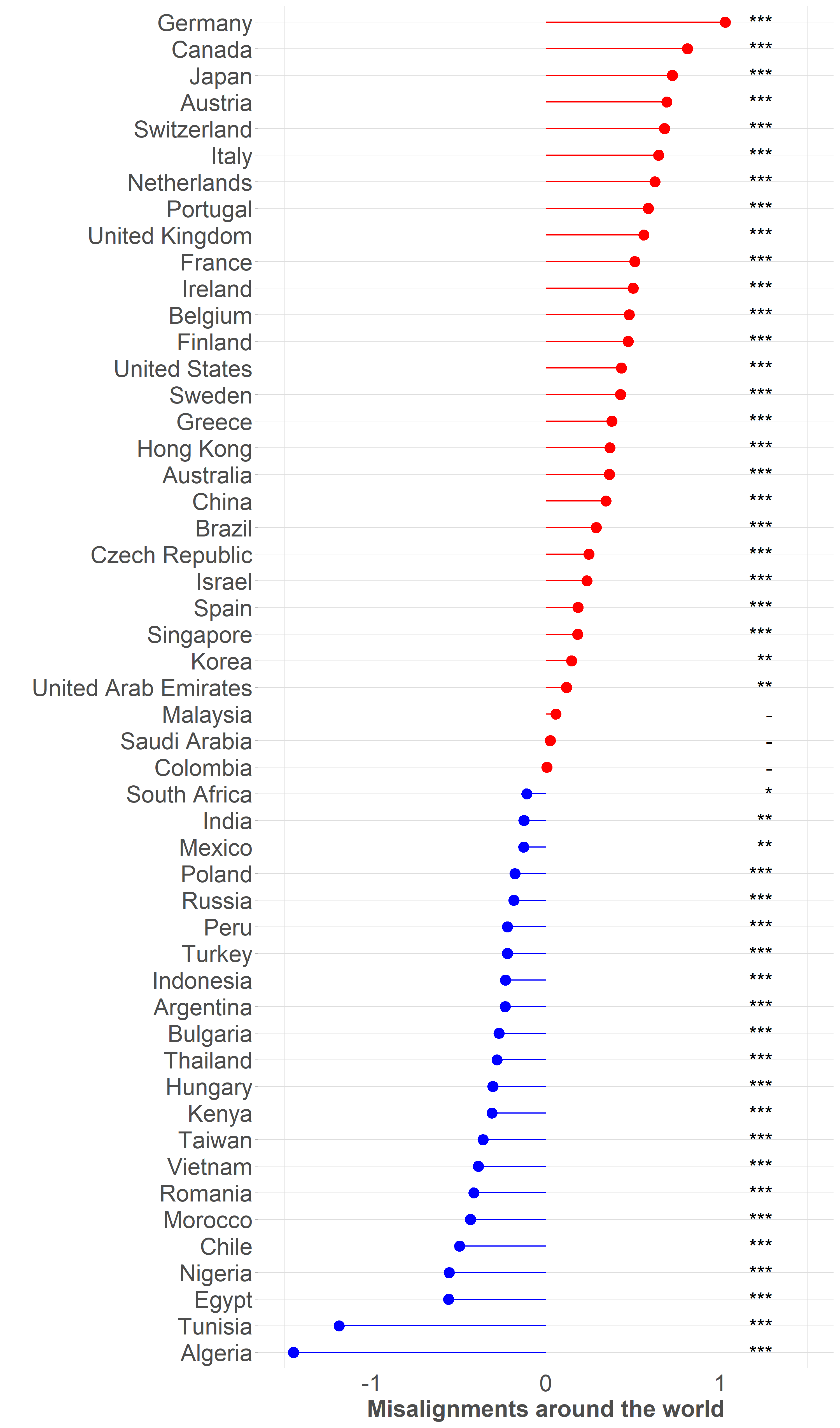


Figure S2. **Degree of conflict misalignments around the world.** Cleveland dot plot of conflict misalignment representing the country-level difference between expected competitiveness (defense investments averaged across all countries except for country *x* against citizens from country *x*) and actual competitiveness (of citizens from country *x* against foreigners, citizens from country *x* excluded). Red lines and dots represent countries where its citizens are less competitive (as measured by attack investments) towards foreigners than foreigners expect them to be; blue lines and dots represent countries where its citizens are more competitive towards foreigners than foreigners expect them to be (as measured by defense investments). Societies are sorted based on *m_x_*. In the right part of the plot, we show the significance level of independent t-tests between participants’ competition in a country and participants’ investments to defend against that particular country (see also SI section 1.7). ****p* < 0.001, ***p* < 0.01, **p* < 0.05, - not significant.

**Table S11.** Independent t-tests; actual competitiveness vs. defense (against) across countries.

| ***Country*** | ***N*_competition_** | ***N*_defense_** | ***t-test*** | ***p-value*** |
| --- | --- | --- | --- | --- |
| Algeria | 5049 | 6257 | 34.035 | <0.0001 |
| Argentina | 5898 | 6179 | 5.3819 | <0.0001 |
| Australia | 6478 | 6180 | -7.5842 | <0.0001 |
| Austria | 6481 | 6179 | -15.075 | <0.0001 |
| Belgium | 5917 | 6153 | -10.766 | <0.0001 |
| Brazil | 6455 | 6126 | -6.2184 | <0.0001 |
| Bulgaria | 6400 | 6235 | 5.6363 | <0.0001 |
| Canada | 7066 | 6186 | -18.508 | <0.0001 |
| Chile | 5978 | 6153 | 10.947 | <0.0001 |
| China | 6226 | 6277 | -7.8385 | <0.0001 |
| Colombia | 5893 | 6205 | -0.001 | 0.9993 |
| Czechia | 6555 | 6163 | -5.0132 | <0.0001 |
| Egypt | 6306 | 6176 | 12.199 | <0.0001 |
| Finland | 7040 | 6104 | -10.525 | <0.0001 |
| France | 5970 | 6153 | -11.378 | <0.0001 |
| Germany | 5913 | 6180 | -21.621 | <0.0001 |
| Greece | 5845 | 6208 | -22.102 | <0.0001 |
| Hong Kong | 6427 | 6152 | -7.9836 | <0.0001 |
| Hungary | 6632 | 6232 | 6.7909 | <0.0001 |
| India | 5741 | 6206 | 3.0253 | 0.0031 |
| Indonesia | 5884 | 6189 | 5.0104 | <0.01 |
| Ireland | 6780 | 6196 | -11.449 | <0.0001 |
| Israel | 6482 | 6169 | -4.8953 | <0.0001 |
| Italy | 6574 | 6207 | -14.797 | <0.0001 |
| Japan | 5767 | 6232 | -15.616 | <0.0001 |
| Kenya | 6343 | 6198 | 6.7425 | <0.0001 |
| Korea | 6638 | 6213 | -3.2229 | <0.01 |
| Malaysia | 6579 | 6197 | -1.1717 | 0.2413 |
| Mexico | 6373 | 6256 | 3.0877 | <0.01 |
| Morocco | 6453 | 6182 | 9.5733 | <0.0001 |
| Netherlands | 6101 | 6208 | -14.375 | <0.0001 |
| Nigeria | 5762 | 6229 | 12.335 | <0.0001 |
| Peru | 6824 | 6172 | 4.9359 | <0.0001 |
| Poland | 6168 | 6168 | 4.0638 | <0.0001 |
| Portugal | 6909 | 6106 | -13.039 | <0.0001 |
| Romania | 6586 | 6187 | 8.8061 | <0.0001 |
| Russia | 6048 | 6208 | 3.8331 | <0.001 |
| Saudi arabia | 6017 | 6159 | -0.1870 | 0.8517 |
| Singapore | 6762 | 6773 | -3.9614 | <0.0001 |
| South Africa | 6455 | 6168 | 2.3964 | 0.0166 |
| Spain | 6482 | 6187 | -4.1772 | <0.0001 |
| Sweden | 6140 | 6132 | -9.108 | <0.0001 |
| Switzerland | 7214 | 6147 | -15.135 | <0.0001 |
| Taiwan | 7400 | 6119 | 8.4605 | <0.0001 |
| Thailand | 7938 | 6102 | 6.6622 | <0.0001 |
| Tunisia | 7631 | 6102 | 29.545 | <0.0001 |
| Turkey | 6891 | 6229 | 5.197 | <0.0001 |
| UAE | 6887 | 6211 | -2.3111 | <0.01 |
| UK | 6683 | 6117 | -12.547 | <0.0001 |
| USA | 5842 | 6195 | -8.5893 | <0.0001 |
| Vietnam | 6993 | 6177 | 8.8081 | <0.0001 |

**1.8. Cross-validation with world value survey**

In this section, we check whether investments in conflict can predict a prominent measure related to conflict in a different dataset (world value survey wave 7). Such cross-validation allows to address two potential concerns. First, we can test whether conflict investments can predict other forms of attitudes related to conflict, such as the willingness to fight for the own country. Second, we cross-validate our findings with a dataset in which participants were recruited with different sampling strategies (such as phone surveys and face to face interviews). To do so, we present two scatterplots showing the relation between either competition or defense from competition and the fraction of people who responded yes to the question “Would you be willing to fight for your country?” as assessed in the world value survey, wave 7 (6). We find that both competition and defense from competition positively associate with the fraction of people who responded yes to this question across countries. Hence, these results lend evidence that conflict investments in the AD-C can predict related attitudes, and are associated to prominent independent datasets that use different stratification strategies.


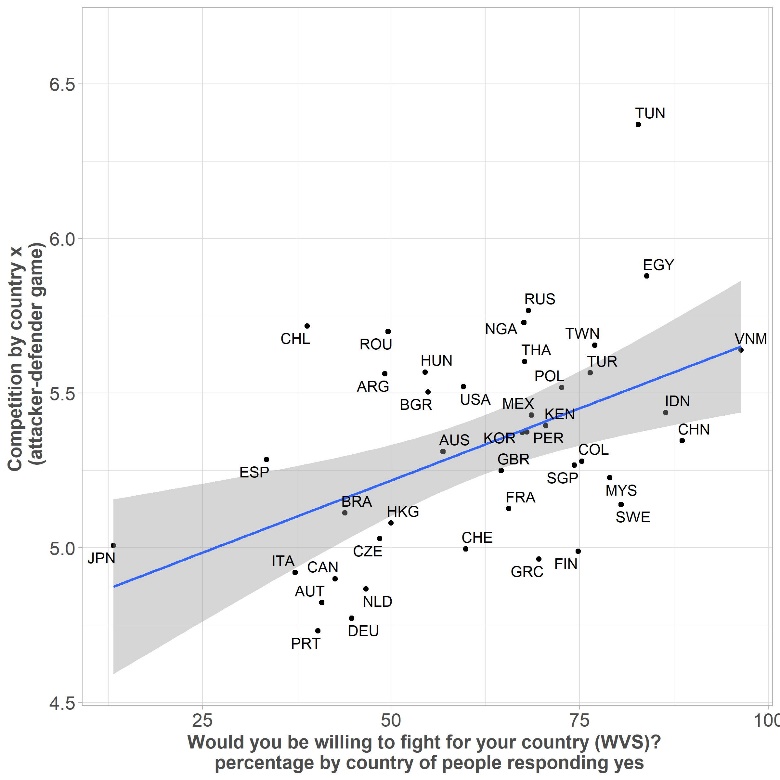


Figure S3. **Competitiveness and willingness to fight for the own country.** Scatterplot showing the relation between the frequency of people who responded yes to the question from the World Value Survey wave 7: “would you be willing to fight for your country?” and competitiveness across-countries in the AD-C.


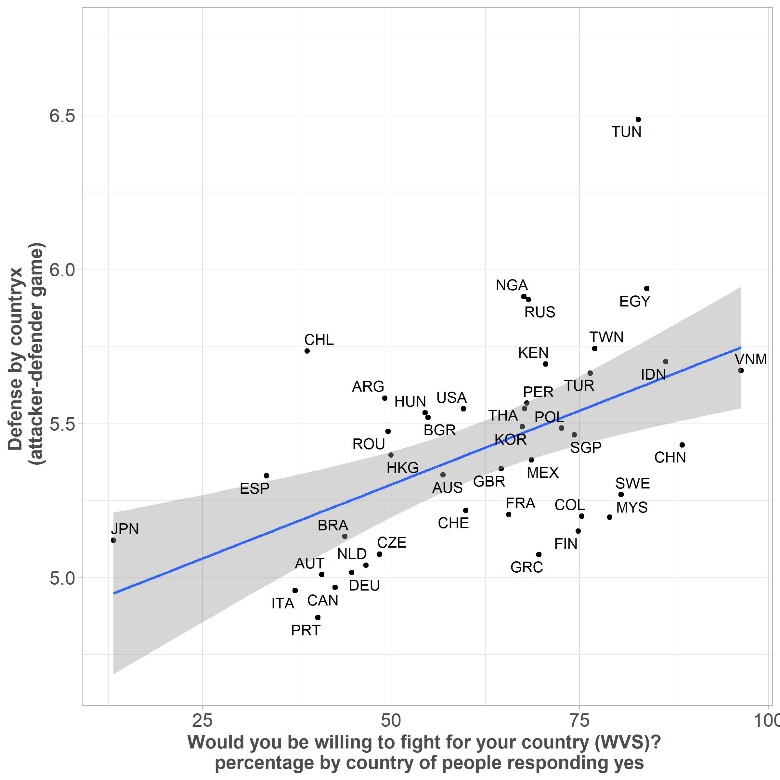


Figure S4. **Defensiveness and willingness to fight for the own country.** Scatterplot showing the relation between the frequency of people who responded yes to the question from the World Value Survey wave 7: “would you be willing to fight for your country?” and defense from competition across countries in the AD-C.

**1.9. Cultural clusters**

A potential variable that might explain variance associated to competitiveness and defense against a country is differences in cultural regions across the world. In this section, we show two plots showing the investment in competition and defense, split by the cultural cluster of the participant and the cultural cluster of the opponent. We clustered nations based on previous seminal work by Inglehart and Baker which plot and categorize nations according to two dimensions: “traditional vs. secular values” and “survival vs. self-expression values” (6). There was a total of eight clusters: African Islamic (United Arab emirates, Algeria, Egypt, Indonesia, Kenya, Morocco, Nigeria, Saudi Arabia, Tunisia, Turkey, South Africa), Catholic Europe (Austria, Belgium, Czech Republic, Spain, France, Hungary, Italy, Poland, Portugal), Confucian (Hong Kong, Japan, Korea, Taiwan), English Speaking (Canada, United Kingdom, Ireland, United States), Latin American (Brazil, Chile, Colombia, Mexico, Peru), Orthodox (Bulgaria, Greece, Romania, Russia), Protestant Europe (Switzerland, Germany, Finland, Netherlands, Sweden), West South Asia (India, Israel, Malaysia, Singapore, Thailand, Vietnam). In general, we observe variation across the cultural clusters in both the competitiveness and defensiveness of participants. We also observe variation in competition and defense based on the cultural cluster of the opponent. That said, it is important to note that, as shown in section 1.11.3, this variation is not significant when considering differences in wealth, institution and historical reputation of conflict.

**
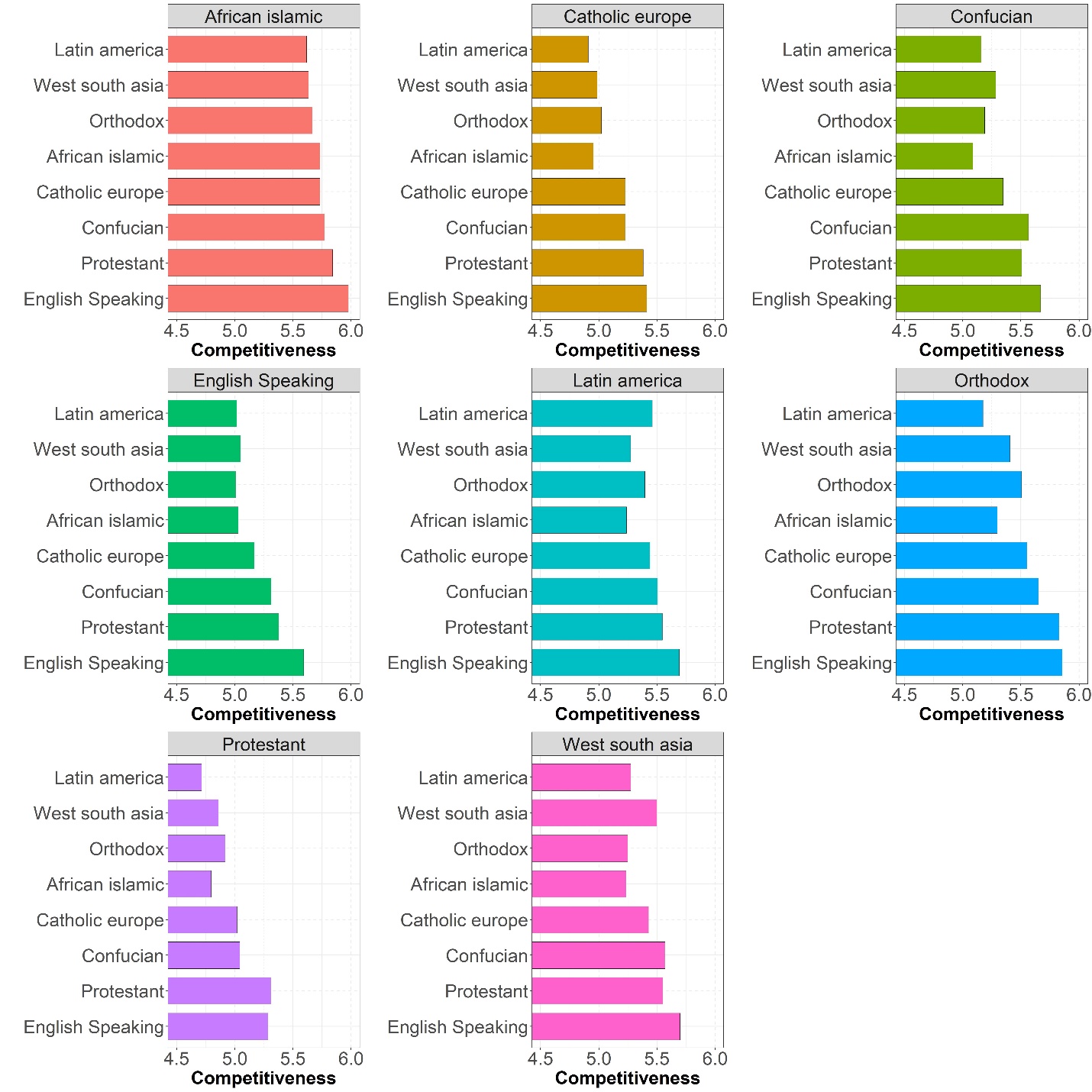
**

Fig S5. **Investment in competitiveness split by cultural cluster of participant and opponent.**


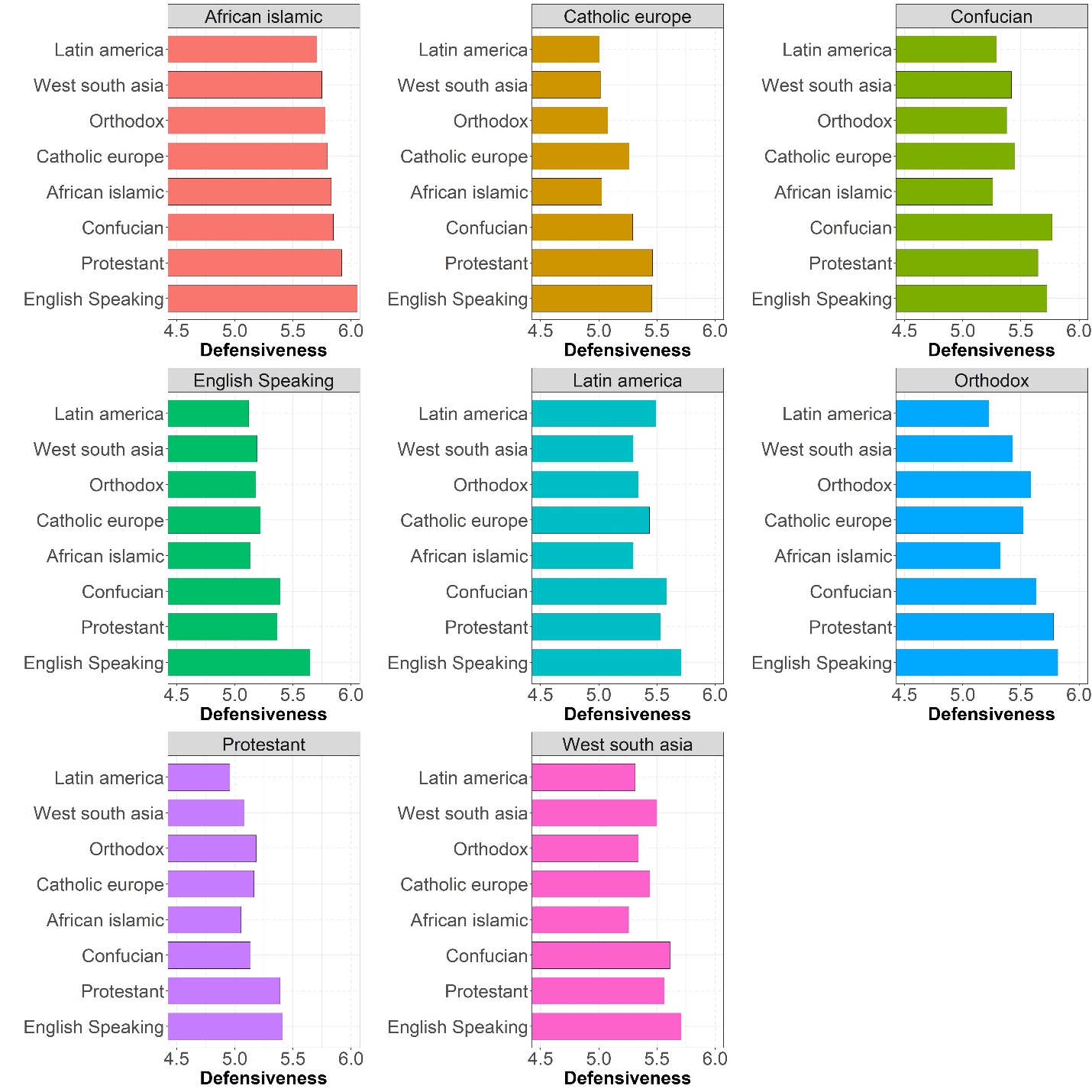


Fig S6. **Investment in defense split by cultural cluster of participant and opponent.**

**1.9.1. West against the rest**

Another potential alternative explanation to the findings shown in Table S5 is that these defense investments are actually driven by a general attitudes towards Western countries, countries that also have a higher reputation of conflict and GDP (7). If that is the case, we would not observe the same results when considering only western countries, or only non-western countries. To address this potential issue, we used the same approach of section 1.5 with the subset of western countries (i.e., protestant, English speaking, and catholic European countries) and the rest. Results remain the same: People invested more in defense when their partners were from richer countries and from countries with a greater reputation of conflict. The findings therefore do not support a simple “west against the rest” explanation.

**Table S12a.** Mixed effect models with cross-societal indicators of opponents predicting defense in conflict from participants belonging to Western countries, only.

| **Defense - West** | ***b*** | ***SE*** | ***t*** | ***p*** |
| --- | --- | --- | --- | --- |
| Gross domestic product - opponent | 0.161 | 0.031 | 5.140 | <0.001 |
| Traditionalism - opponent | -0.011 | 0.021 | -0.536 | 0.594 |
| Institutional quality - opponent | -0.004 | 0.020 | -0.204 | 0.839 |
| Ecological stress - opponent | -0.023 | 0.019 | -1.259 | 0.214 |
| Reputation of conflict - opponent | 0.106 | 0.016 | 6.529 | <0.001 |
| *N_countries_* | 19 |  |  |  |

*Notes.* Tests are two-sided. *N*_observations_= 118,375; *N*_subjects_ = 4831; *N*_country-opponent_ = 51.

**Table S12b.** Mixed effect models with cross-societal indicators of opponents predicting defense in conflict from participants belonging to non-Western countries, only

| **Defense - Non-west** | ***b*** | ***SE*** | ***t*** | ***p*** |
| --- | --- | --- | --- | --- |
| Gross domestic product - opponent | 0.100 | 0.029 | 3.400 | 0.001 |
| Traditionalism - opponent | -0.004 | 0.02 | -0.195 | 0.846 |
| Institutional quality - opponent | 0.020 | 0.019 | 1.031 | 0.308 |
| Ecological stress - opponent | -0.008 | 0.017 | -0.471 | 0.640 |
| Reputation of conflict - opponent | 0.103 | 0.015 | 6.774 | <0.001 |
| *N_countries_* | 32 |  |  |  |

*Notes.* Tests are two-sided. *N*_observations_= 196,899; *N*_subjects_ = 8032; *N*_country-opponent_ = 51.

**1.10.** **Cross-societal models without imputation**

A potential concern from the model presented in Table S5 and Table S6 is that results from these models may be a statistical artifact due to the fact that we replaced missing cases with multiple imputation techniques. Therefore, in this section, we present a robustness check with country-level regressions of the cross-societal indicators predicting defense against foreigners and competition without the use of imputation techniques (Table S13 and S14). Due to the missing cases such models report results of a subset of countries (38 out of 51). When including all cross-societal indicators in one model, such models drop all observations that contain at least one missing case in one of the indicators. Therefore, we also present the individual correlations of each indicator with both defense and competition (Table S15). Such independent correlations contain all available observations for each indicator (see Table S16). Overall, the interpretation of the results remains the same. Gross domestic product and reputation of conflict remain the best predictors of defense against country *x* in both regression and correlation tables (see Table S13 and Table S15). Although with this analytic strategy the institutional quality indicator becomes marginally significant (*p* = .07), it remains the best predictor of actual competitiveness by country *x* (Table S14, *r* = -0.665).

**Table S13.** Regression model of gross domestic product, institutional quality, reputation of conflict, traditionalism, and ecological stress (without imputation) predicting defense against country *x*.

| **Defense against country *x*** | ***b*** | ***SE*** | ***t*** | ***p*** |
| --- | --- | --- | --- | --- |
| Gross domestic product | 0.150 | 0.038 | 3.944 | <0.001 |
| Institutional quality | 0.009 | 0.023 | 0.399 | 0.693 |
| Reputation of conflict | 0.092 | 0.017 | 5.446 | <0.001 |
| Traditionalism | 0.010 | 0.021 | 0.478 | 0.636 |
| Ecological stress | 0.005 | 0.037 | 0.127 | 0.900 |

*Notes.* Tests are two-sided. *N*_observations_ = 38.

**Table S14.** Regression model of gross domestic product, institutional quality, reputation of conflict, traditionalism, and ecological stress (without imputation) predicting competition by country *x*.

| **Competition by country *x*** | ***b*** | ***SE*** | ***t*** | ***p*** |
| --- | --- | --- | --- | --- |
| Gross domestic product | -0.036 | 0.095 | -0.355 | 0.725 |
| Institutional quality | -0.106 | 0.058 | -1.842 | 0.075 |
| Reputation of conflict | 0.031 | 0.042 | 0.736 | 0.467 |
| Traditionalism | -0.031 | 0.053 | -0.581 | 0.566 |
| Ecological stress | -0.061 | 0.093 | -0.655 | 0.517 |

*Notes.* Tests are two-sided. *N*_observations_ = 38.

**Table S15.** Independent correlation estimates of cross societal indicator with defense against country *x* and actual competitiveness.

|  | **Defense against country *x*** | | **Competition by country *x*** | |
| --- | --- | --- | --- | --- |
| **Indicator** | ***r*** | ***p*** | ***r*** | ***p*** |
| GDP p/c | 0.699 | <0.001 | -0.590 | <0.001 |
| Institutional quality | 0.568 | <0.001 | -0.665 | <0.001 |
| Reputation of conflict | 0.467 | <0.001 | 0.032 | 0.829 |
| Ecological stress | -0.395 | 0.007 | 0.407 | 0.005 |
| Cultural orientation | 0.426 | 0.003 | -0.388 | 0.008 |

*Notes.* See Table S16 for number the of observations for each indicator.

**1.11. Cross-societal models with additional indicators**

Another potential concern of the findings discussed in the manuscript is that results may be driven by the selection of the specific indicators used in the main analyses. To address this potential issue, in this section we provide several models that test the robustness of the findings presented in section 1.3 with additional cross-societal indicators. First, we add several indicators to the operationalization of wealth, institutions, and ecological stress (section 1.11.1). Second, in section 1.11.2 we consider three robustness checks for the reputation of conflict: log-transformation, reputation of conflict based on recent disputes (50 years ago), and reputation of conflict based on distant disputes (more than 50 years ago). Finally, we also provide a robustness check for cultural orientation by using a prominent classification of countries based on their cultural values (section 1.11.13). In sum, these robustness checks replicate the findings presented in the manuscript.

**1.11.1. PCA institutions, ecology, and wealth**

As an additional robustness check, we retrieved additional indicators for institutions, ecological stress, and wealth. To better represent an underlying dimension common across all potential indicators, we used a bottom-up approach. First, we retrieved several potential indicators related to institutions (*rule of law, government effectiveness, democracy, corruption, global competitiveness*), ecological stress (*historical prevalence of pathogens, vulnerability to natural disasters, average tuberculosis per 10,000 people, death by communicable disease*) and wealth (*shadow economy, gross national income, gross domestic product, historical gross domestic product*). We did not use the same approach for reputation of conflict or cultural orientation because either, to our knowledge, there are not multiple versions of such indicators (reputation of conflict) or the indicator was already the result of a factor analysis comprising several cultural

**Table S16.** Summary of indicators, their different operationalizations, source, years, and available observations.

| ***Indicators*** | ***Source*** | ***Year*** | ***N*** |
| --- | --- | --- | --- |
| *Cultural orientation* |  |  |  |
| Traditional vs. secular values  Cultural clusters | World value survey (WVS)  World value survey (WVS) | 1981-2014  1981-2014 | 45  51 |
| *Reputation of conflict* |  |  |  |
| Prevalence of historical conflict  Recent historical conflict  Distant historical conflict | Correlates of war  Correlates of war (<50 years ago)  Correlates of war (>50 years ago) | 1816-2007  1816-2007  1816-2007 | 49  49  49 |
| *Ecological Stress* |  |  |  |
| Historical prevalence of pathogens | Murray. D. R.. & Schaller. M. (8). | 2010 | 47 |
| Vulnerability to natural disasters  Average tuberculosis per 10,000  Death by communicable disease | Environmental sustainability index  World health organization  World health organization | 2005  2017  2017 | 45  50  50 |
| *Economic wealth* |  |  |  |
| GDP per capita  Gross national income (GNI)  Historical GDP 1950  Shadow economy | World bank  World bank  Maddison project (9)  World bank | 2019  2019  2013  2019 | 50  50  49  49 |
| *Quality of institutions* |  |  |  |
| Government effectiveness | World bank | 2017 | 51 |
| Rule of law | Freedom house | 2018 | 51 |
| Democracy  Corruption  Global competitiveness | Economist intelligence unit  World bank  World economic forum | 2017  2017  2018 | 51  51  47 |

values (cultural orientation). However, we ran different types of robustness checks for both reputation and cultural orientation (see section 1.11.2 and 1.11.3). Then, using the package *MissForest* we used imputation techniques to fill missing cases (complete observations by indicator are shown in Table S16). After that, we ran a principal component analysis to extract components that better explain variance across the selected indicators. We selected the number of components that explained 80 percent of the variance. For wealth, we retrieved two components (PCA Wealth 1 and PCA Wealth 2). PCA Wealth 1 mostly loaded on GDP, GNI, and shadow economy. It negatively correlated with GDP per capita, gross national income, and historical GDP, while it is positively correlated with the shadow economy indicator. Thus, this indicator can be interpreted a proxy of the current economic situation. PCA Wealth 2 is a component that mostly loaded on historical GDP, and therefore can be interpreted as a proxy of historical wealth. Institutional indicators loaded on one component (PCA Institutions) that positively correlates with all the cross-societal indicators related to institutions. Ecological stress explained 80% of variance with two components. PCA stress 1 is positively correlated with all cross-societal indicators related to ecological stress. PCA stress 2 negatively correlates with prevalence of infectious diseases and vulnerability to natural disasters (thereby also justifying the choice of the indicator in the main analyses), and positively correlate with average tuberculosis per 10,000 people, death by communicable disease*.* Finally, we ran multiple regression models predicting expected and actual competitiveness (Table S17 and Table S18). Results remained the same. The current economic situation (PCA wealth 1) and reputation of conflict were still significant predictors of defense against foreigners, while institutions remained significant predictors of competition against foreigners.

**Table S17.** Regression model of wealth (1 and 2), institution, reputation of conflict, cultural orientation, and ecological stress (1 and 2) predicting defense against country *x*.

| **Defense against country *x*** | ***b*** | ***SE*** | ***t*** | ***p*** |
| --- | --- | --- | --- | --- |
| PCA Wealth 1 | -0.073 | 0.02 | -3.593 | 0.001 |
| PCA Wealth 2 | 0.003 | 0.016 | 0.158 | 0.875 |
| PCA Institutions | 0.007 | 0.017 | 0.411 | 0.683 |
| Reputation of conflict | 0.095 | 0.015 | 6.307 | <0.001 |
| Cultural orientation | -0.007 | 0.021 | -0.348 | 0.730 |
| PCA Stress 1 | -0.022 | 0.013 | -1.674 | 0.101 |
| PCA Stress 2 | 0.014 | 0.014 | 1.019 | 0.314 |

*Notes.* Tests are two-sided. *N*_observations_ = 51.

**Table S18.** Regression model of wealth (1 and 2), institution, reputation of conflict, cultural orientation, and ecological stress (1 and 2) predicting competition by country *x*.

| **Competition by country *x*** | ***b*** | ***SE*** | ***t*** | ***p*** |
| --- | --- | --- | --- | --- |
| PCA Wealth 1 | -0.002 | 0.062 | -0.038 | 0.970 |
| PCA Wealth 2 | 0.013 | 0.05 | 0.256 | 0.799 |
| PCA Institutions | -0.119 | 0.051 | -2.339 | 0.024 |
| Reputation of conflict | -0.010 | 0.046 | -0.209 | 0.836 |
| Cultural orientation | -0.041 | 0.062 | -0.652 | 0.518 |
| PCA Stress 1 | -0.025 | 0.039 | -0.644 | 0.523 |
| PCA Stress 2 | -0.082 | 0.042 | -1.937 | 0.059 |

*Notes.* Tests are two-sided. *N*_observations_ = 51.

**1.11.1.1 Potential outliers: Algeria and Tunisia**

From Figure 2B, it looks like that there are two potential outliers in conflict misalignments. These two countries (Algeria and Tunisia) seem to be perceived as less competitive than warranted to a greater extent than the rest of the other countries. Therefore, as a further robustness test to check that our results were not driven by these two countries, we also conducted additional analyses in which we removed these two countries. First, we computed the correlation between competition by country *x* and defense against country *x* without Algeria and Tunisia, and found again a negative correlation between the two (*r* = 0.39, *p* = 0.006, see Figure S7). Second, we computed cross-societal models excluding these two countries. As shown in Table S19 and Table S20, this did not change the results. The quality of institutions remains the best predictor of actual competitiveness against foreigners while reputation of conflict and wealth (PCA wealth 1) remain the main indicators predicting defense against competition by foreigners.

**
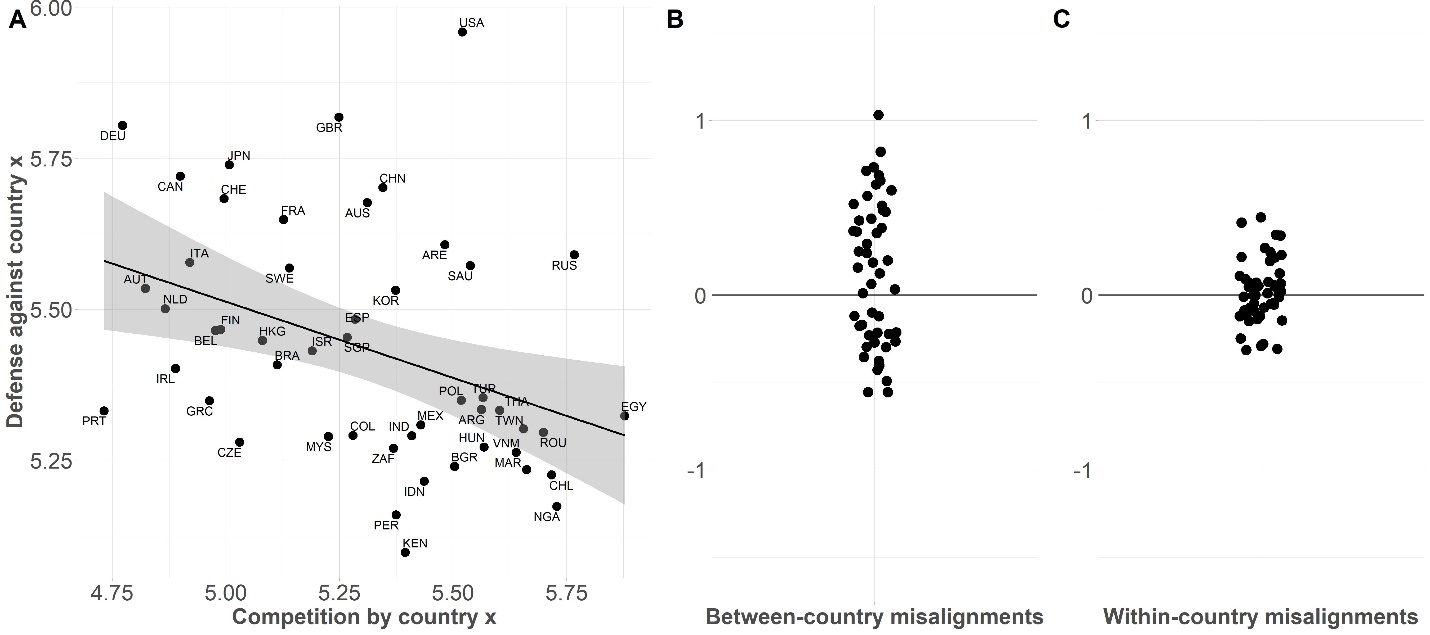
**

Fig. S7**. Figure 2 without outliers.** Replication of figure 2 of the manuscript excluding participants from Algeria and Tunisia.

**Table S19.** Regression model of wealth (1 and 2), institution, reputation of conflict, cultural orientation, and ecological stress (1 and 2) predicting defense against country *x*.

| **Defense against country *x*** | ***b*** | ***SE*** | ***t*** | ***p*** |
| --- | --- | --- | --- | --- |
| PCA Wealth 1 | -0.074 | 0.021 | -3.456 | 0.001 |
| PCA Wealth 2 | 0.004 | 0.017 | 0.213 | 0.832 |
| PCA Institutions | 0.007 | 0.018 | 0.411 | 0.683 |
| Reputation of conflict | 0.002 | 0.001 | 6.149 | <0.001 |
| Cultural orientation | -0.007 | 0.021 | -0.308 | 0.760 |
| PCA Stress 1 | -0.021 | 0.013 | -1.609 | 0.115 |
| PCA Stress 2 | 0.015 | 0.014 | 1.031 | 0.309 |

*Notes.* Tests are two-sided. *N*_observations_ = 49.

**Table S20.** Regression model of wealth (1 and 2), institution, reputation of conflict, cultural orientation, and ecological stress (1 and 2) predicting competition by country *x*.

| **Competition by country *x*** | ***b*** | ***SE*** | ***t*** | ***p*** |
| --- | --- | --- | --- | --- |
| PCA Wealth 1 | -0.024 | 0.048 | -0.493 | 0.625 |
| PCA Wealth 2 | 0.057 | 0.039 | 1.474 | 0.148 |
| PCA Institutions | -0.109 | 0.04 | -2.747 | 0.009 |
| Reputation of conflict | 0.001 | 0.001 | 0.519 | 0.607 |
| Cultural orientation | -0.013 | 0.048 | -0.28 | 0.781 |
| PCA Stress 1 | -0.012 | 0.03 | -0.408 | 0.685 |
| PCA Stress 2 | -0.055 | 0.033 | -1.693 | 0.098 |

*Notes.* Tests are two-sided. *N*_observations_= 49.

**1.11.2. Log reputation of conflict, recent and distant reputation of conflict**

In this section, we provide a robustness check of historical reputation. We ran the same models presented in Table S17 and S18 with different version of the reputation of conflict variable. First, we applied a log transformation of reputation of conflict (Table S21 and S22). Then, we created a new variable of reputation of conflict that only considered conflict among nations that happened more than 50 years ago (Table S23, Table S24). Finally, we considered all models with a reputation of conflict that comprises disputes that happened in the last 50 years, only (Table S25, and S26). Results remained the same: history of reputation only matters for defense against foreigners (together with wealth PC1), and is not significantly associated with actual competition (competition by country *x*).

**Table S21.** Regression model of wealth (1 and 2), institution, log reputation of conflict, cultural orientation, and ecological stress (1 and 2) predicting defense against country *x*.

| **Defense against country *x*** | ***b*** | ***SE*** | ***t*** | ***p*** |
| --- | --- | --- | --- | --- |
| PCA Wealth 1 | -0.103 | 0.023 | -4.511 | <0.001 |
| PCA Wealth 2 | -0.006 | 0.019 | -0.326 | 0.746 |
| PCA Institutions | -0.008 | 0.018 | -0.453 | 0.653 |
| Log(reputation of conflict) | 0.086 | 0.017 | 5.028 | <0.001 |
| Cultural orientation | -0.011 | 0.023 | -0.458 | 0.649 |
| PCA Stress 1 | -0.023 | 0.014 | -1.601 | 0.117 |
| PCA Stress 2 | 0.02 | 0.016 | 1.279 | 0.208 |

*Notes.* Tests are two-sided. *N*_observations_ = 51.

**Table S22.** Regression model of wealth (1 and 2), institution, log reputation of conflict, cultural orientation, and ecological stress (1 and 2) predicting competition by country *x*.

| **Competition by country *x*** | ***b*** | ***SE*** | ***t*** | ***p*** |
| --- | --- | --- | --- | --- |
| PCA Wealth 1 | 0.018 | 0.061 | 0.29 | 0.773 |
| PCA Wealth 2 | 0.036 | 0.05 | 0.722 | 0.474 |
| PCA Institutions | -0.123 | 0.048 | -2.535 | 0.015 |
| Log(reputation of conflict) | -0.078 | 0.046 | -1.708 | 0.095 |
| Cultural orientation | -0.009 | 0.062 | -0.143 | 0.887 |
| PCA Stress 1 | -0.034 | 0.038 | -0.891 | 0.378 |
| PCA Stress 2 | -0.093 | 0.041 | -2.252 | 0.029 |

*Notes.* Tests are two-sided. *N*_observations_ = 51.

**Table S23.** Regression model of wealth (1 and 2), institution, distant reputation of conflict, cultural orientation, and ecological stress (1 and 2) predicting defense against country *x*.

| **Defense against country *x*** | ***b*** | ***SE*** | ***t*** | ***p*** |
| --- | --- | --- | --- | --- |
| PCA Wealth 1 | -0.077 | 0.02 | -3.932 | <0.001 |
| PCA Wealth 2 | 0.006 | 0.016 | 0.394 | 0.695 |
| PCA Institutions | 0.002 | 0.016 | 0.153 | 0.879 |
| Reputation of conflict - distant | 0.003 | 0.001 | 6.728 | <0.001 |
| Cultural orientation | -0.007 | 0.02 | -0.334 | 0.740 |
| PCA Stress 1 | -0.015 | 0.013 | -1.162 | 0.252 |
| PCA Stress 2 | 0.012 | 0.014 | 0.883 | 0.382 |

*Notes.* Tests are two-sided. *N*_observations_ = 51.

**Table S24.** Regression model of wealth (1 and 2), institution, distant reputation of conflict, cultural orientation, and ecological stress (1 and 2) predicting competition by country *x*.

| **Competition by country *x*** | ***b*** | ***SE*** | ***t*** | ***p*** |
| --- | --- | --- | --- | --- |
| PCA Wealth 1 | -0.002 | 0.062 | -0.038 | 0.970 |
| PCA Wealth 2 | 0.014 | 0.049 | 0.284 | 0.778 |
| PCA Institutions | -0.121 | 0.051 | -2.39 | 0.021 |
| Reputation of conflict - distant | -0.001 | 0.001 | -0.444 | 0.659 |
| Cultural orientation | -0.037 | 0.062 | -0.596 | 0.554 |
| PCA Stress 1 | -0.028 | 0.04 | -0.704 | 0.485 |
| PCA Stress 2 | -0.082 | 0.042 | -1.952 | 0.057 |

*Notes.* Tests are two-sided. *N*_observations_ = 51.

**Table S25.** Regression model of wealth (1 and 2), institution, recent reputation of conflict, cultural orientation, and ecological stress (1 and 2) predicting defense against country *x*.

| **Defense against country *x*** | ***b*** | ***SE*** | ***t*** | ***p*** |
| --- | --- | --- | --- | --- |
| PCA Wealth 1 | -0.07 | 0.024 | -2.942 | 0.005 |
| PCA Wealth 2 | 0.004 | 0.019 | 0.207 | 0.837 |
| PCA Institutions | 0.006 | 0.02 | 0.324 | 0.747 |
| Reputation of conflict - recent | 0.003 | 0.001 | 4.318 | <0.001 |
| Cultural orientation | 0.002 | 0.024 | 0.102 | 0.919 |
| PCA Stress 1 | -0.035 | 0.015 | -2.328 | 0.025 |
| PCA Stress 2 | 0.015 | 0.016 | 0.948 | 0.349 |

*Notes.* Tests are two-sided. *N*_observations_= 51.

**Table S26.** Regression model of wealth (1 and 2), institution, recent reputation of conflict, cultural orientation, and ecological stress (1 and 2) predicting competition by country *x*.

| **Competition by country *x*** | ***b*** | ***SE*** | ***t*** | ***p*** |
| --- | --- | --- | --- | --- |
| PCA Wealth 1 | 0.001 | 0.062 | 0.002 | 0.998 |
| PCA Wealth 2 | 0.008 | 0.05 | 0.169 | 0.866 |
| PCA Institutions | -0.114 | 0.052 | -2.217 | 0.032 |
| Reputation of conflict - recent | 0.001 | 0.002 | 0.225 | 0.823 |
| Cultural orientation | -0.048 | 0.062 | -0.77 | 0.445 |
| PCA Stress 1 | -0.024 | 0.039 | -0.623 | 0.537 |
| PCA Stress 2 | -0.08 | 0.043 | -1.882 | 0.067 |

*Notes.* Tests are two-sided. *N*_observations_ = 51.

**1.11.3. Cultural clusters**

In this section, we provide an additional robustness check for the cultural orientation results (traditionalism vs. secularism) by using cultural clusters. Instead of assessing the effect of cultural values that are hypothesized to be related with conflict (i.e., traditionalism), we divided countries across cultural cluster based on the world value survey (6). As cultural cluster is a categorical variable with 8 categories (see section 1.9), instead of regressions we performed an ANOVA. Results remain the same (Table S27 and Table S28). While wealth and reputation of conflict explained significant fraction of variance for defense against foreigners (defense against country *x*), institutions explained a significant fraction of variance of actual competitiveness (competition by country *x*). The effect of cultural cluster was not significant for both competition and defense. Contrary to other cross-societal analyses, we found that PCA Stress 1 and 2 also explained a significant portion of variance for actual competitiveness. That said, as this pattern only emerged with this set of specifications, we should not derive strong conclusion from these results.

**Table S27.** ANOVA of cross-societal indicators predicting defense against country *x*.

| **Defense against country *x*** | ***Df*** | ***Sum Sq*** | ***Mean Sq*** | ***F value*** | ***Pr(>F)*** |
| --- | --- | --- | --- | --- | --- |
| PCA Wealth 1 | 1 | 1.027 | 1.027 | 103 | <0.001 |
| PCA Wealth 2 | 1 | 0.001 | 0.001 | 0.031 | 0.861 |
| PCA Institutions | 1 | 0.001 | 0.001 | 0.029 | 0.865 |
| Reputation of conflict | 1 | 0.448 | 0.448 | 44.967 | <0.001 |
| Cultural orientation - clusters | 7 | 0.070 | 0.010 | 1.004 | 0.444 |
| PCA Stress 1 | 1 | 0.016 | 0.016 | 1.641 | 0.208 |
| PCA Stress 2 | 1 | 0.010 | 0.010 | 0.959 | 0.334 |

*Notes.* Tests are two-sided. *N*_observations_ = 51.

**Table S28.** ANOVA of cross-societal indicators predicting competition by country *x*.

| **Competition by country *x*** | ***Df*** | ***Sum Sq*** | ***Mean Sq*** | ***F value*** | ***Pr(>F)*** |
| --- | --- | --- | --- | --- | --- |
| PCA Wealth 1 | 1 | 2.315 | 2.315 | 32.08 | 0.001 |
| PCA Wealth 2 | 1 | 0.052 | 0.052 | 0.715 | 0.403 |
| PCA Institutions | 1 | 0.432 | 0.432 | 5.989 | 0.019 |
| Reputation of conflict | 1 | 0.001 | 0.001 | 0.006 | 0.938 |
| Cultural orientation - clusters | 7 | 0.824 | 0.118 | 1.631 | 0.157 |
| PCA Stress 1 | 1 | 0.508 | 0.508 | 7.033 | 0.012 |
| PCA Stress 2 | 1 | 0.278 | 0.278 | 3.846 | 0.057 |

*Notes.* Tests are two-sided. *N*_observations_ = 51.

**1.11.4. Democracy**

As additional cross-validation of the quality of institutions results, we retrieved secondary data from a dataset that assesses the quality of institutions by investigating different aspects of democracy: https://v-dem.net/. In particular, this dataset distinguishes between high-level principles of democracy: deliberative, electoral, liberal, participatory, and egalitarian (10). We then retrieved five indicators representing each of these aspects of democracies (year 2021). We ran two kind of robustness checks. First, we checked whether each of these measures correlated with the quality of institutions measure presented in the main analyses of the paper. We found high between-country correlations between the quality of institutions measure and the democracy indicators (0.69 < *r* < 0.80), suggesting that the selected set of indicators to assess quality of institutions nicely captures between-country variation. Second, we again ran the cross-societal models without imputation (see section 1.10), replacing the quality of institutions indicator with each indicator of democracy. We found that results remain the same. Wealth indicators and history of conflict remain the only significant predictors of defense against foreigners from a particular country (see Table S29 to Table S33), while institutional indicators (in this case, five democracy indicators) remains the only significant predictor of competition against foreigners (see Table S34 to Table S38).

**Table S29.** Regression model of gross domestic product, institutional quality, reputation of conflict, traditionalism, and ecological stress (without imputation) predicting defense against country *x*.

| **Defense against country *x*** | ***b*** | ***SE*** | ***t*** | ***p*** |
| --- | --- | --- | --- | --- |
| Gross domestic product | 0.162 | 0.027 | 5.931 | <0.001 |
| Deliberative democracy | 0.003 | 0.091 | 0.031 | 0.975 |
| Reputation of conflict | 0.089 | 0.016 | 5.444 | <0.001 |
| Traditionalism | 0.013 | 0.02 | 0.658 | 0.515 |
| Ecological stress | 0.005 | 0.037 | 0.131 | 0.896 |

*Notes.* Tests are two-sided. *N*_observations_ = 38.

**Table S30.** Regression model of gross domestic product, institutional quality, reputation of conflict, traditionalism, and ecological stress (without imputation) predicting defense against country *x*.

| **Defense against country *x*** | ***b*** | ***SE*** | ***t*** | ***p*** |
| --- | --- | --- | --- | --- |
| Gross domestic product | 0.167 | 0.026 | 6.487 | <.001 |
| Electoral democracy | -0.028 | 0.087 | -0.315 | 0.755 |
| Reputation of conflict | 0.087 | 0.016 | 5.451 | <.001 |
| Traditionalism | 0.014 | 0.02 | 0.686 | 0.497 |
| Ecological stress | 0.004 | 0.037 | 0.115 | 0.909 |

*Notes.* Tests are two-sided. *N*_observations_ = 38.

**Table S31.** Regression model of gross domestic product, institutional quality, reputation of conflict, traditionalism, and ecological stress (without imputation) predicting defense against country *x*.

| **Defense against country *x*** | ***b*** | ***SE*** | ***t*** | ***p*** |
| --- | --- | --- | --- | --- |
| Gross domestic product | 0.169 | 0.028 | 6.069 | <.001 |
| Liberal democracy | -0.032 | 0.093 | -0.346 | 0.732 |
| Reputation of conflict | 0.087 | 0.016 | 5.297 | <.001 |
| Traditionalism | 0.014 | 0.02 | 0.715 | 0.48 |
| Ecological stress | 0.005 | 0.037 | 0.135 | 0.894 |

*Notes.* Tests are two-sided. *N*_observations_ = 38.

**Table S32.** Regression model of gross domestic product, institutional quality, reputation of conflict, traditionalism, and ecological stress (without imputation) predicting defense against country *x*.

| **Defense against country *x*** | ***b*** | ***SE*** | ***t*** | ***p*** |
| --- | --- | --- | --- | --- |
| Gross domestic product | 0.171 | 0.028 | 6.153 | <.001 |
| Participatory democracy | -0.059 | 0.124 | -0.479 | 0.635 |
| Reputation of conflict | 0.086 | 0.016 | 5.352 | <.001 |
| Traditionalism | 0.014 | 0.02 | 0.686 | 0.498 |
| Ecological stress | 0.004 | 0.037 | 0.112 | 0.912 |

*Notes.* Tests are two-sided. *N*_observations_ = 38.

**Table S33.** Regression model of gross domestic product, institutional quality, reputation of conflict, traditionalism, and ecological stress (without imputation) predicting defense against country *x*.

| **Defense against country *x*** | ***b*** | ***SE*** | ***t*** | ***p*** |
| --- | --- | --- | --- | --- |
| Gross domestic product | 0.162 | 0.029 | 5.640 | <.001 |
| Egalitarian democracy | 0.001 | 0.114 | 0.001 | 0.999 |
| Reputation of conflict | 0.089 | 0.017 | 5.351 | <.001 |
| Traditionalism | 0.013 | 0.021 | 0.642 | 0.526 |
| Ecological stress | 0.005 | 0.037 | 0.133 | 0.895 |

*Notes.* Tests are two-sided. *N*_observations_ = 38.

**Table S34.** Regression model of gross domestic product, institutional quality, reputation of conflict, traditionalism, and ecological stress (without imputation) predicting competition by country *x*.

| **Competition by country *x*** | ***b*** | ***SE*** | ***t*** | ***p*** |
| --- | --- | --- | --- | --- |
| Gross domestic product | -0.08 | 0.066 | -1.207 | 0.236 |
| Deliberative democracy | -0.52 | 0.22 | -2.362 | 0.024 |
| Reputation of conflict | 0.03 | 0.04 | 0.744 | 0.462 |
| Traditionalism | -0.047 | 0.049 | -0.964 | 0.343 |
| Ecological stress | -0.051 | 0.09 | -0.565 | 0.576 |

*Notes.* Tests are two-sided. *N*_observations_ = 38.

**Table S35.** Regression model of gross domestic product, institutional quality, reputation of conflict, traditionalism, and ecological stress (without imputation) predicting competition by country *x*.

| **Competition by country *x*** | ***b*** | ***SE*** | ***t*** | ***p*** |
| --- | --- | --- | --- | --- |
| Gross domestic product | -0.093 | 0.063 | -1.479 | 0.149 |
| Electoral democracy | -0.511 | 0.212 | -2.408 | 0.022 |
| Reputation of conflict | 0.035 | 0.039 | 0.892 | 0.379 |
| Traditionalism | -0.064 | 0.048 | -1.336 | 0.191 |
| Ecological stress | -0.076 | 0.09 | -0.845 | 0.405 |

*Notes.* Tests are two-sided. *N*_observations_ = 38.

**Table S36.** Regression model of gross domestic product, institutional quality, reputation of conflict, traditionalism, and ecological stress (without imputation) predicting competition by country *x*.

| **Competition by country *x*** | ***b*** | ***SE*** | ***t*** | ***p*** |
| --- | --- | --- | --- | --- |
| Gross domestic product | -0.078 | 0.068 | -1.142 | 0.262 |
| Liberal democracy | -0.524 | 0.229 | -2.292 | 0.029 |
| Reputation of conflict | 0.031 | 0.04 | 0.767 | 0.449 |
| Traditionalism | -0.054 | 0.049 | -1.104 | 0.278 |
| Ecological stress | -0.063 | 0.091 | -0.696 | 0.492 |

*Notes.* Tests are two-sided. *N*_observations_ = 38.

**Table S37.** Regression model of gross domestic product, institutional quality, reputation of conflict, traditionalism, and ecological stress (without imputation) predicting competition by country *x*.

| **Competition by country *x*** | ***b*** | ***SE*** | ***t*** | ***p*** |
| --- | --- | --- | --- | --- |
| Gross domestic product | -0.078 | 0.068 | -1.146 | 0.26 |
| Participatory democracy | -0.686 | 0.304 | -2.261 | 0.031 |
| Reputation of conflict | 0.035 | 0.039 | 0.877 | 0.387 |
| Traditionalism | -0.066 | 0.048 | -1.367 | 0.181 |
| Ecological stress | -0.073 | 0.091 | -0.803 | 0.428 |

*Notes.* Tests are two-sided. *N*_observations_ = 38.

**Table S38.** Regression model of gross domestic product, institutional quality, reputation of conflict, traditionalism, and ecological stress (without imputation) predicting competition by country *x*.

| **Competition by country *x*** | ***b*** | ***SE*** | ***t*** | ***p*** |
| --- | --- | --- | --- | --- |
| Gross domestic product | -0.05 | 0.068 | -0.739 | 0.465 |
| Egalitarian democracy | -0.749 | 0.268 | -2.789 | 0.009 |
| Reputation of conflict | 0.02 | 0.039 | 0.511 | 0.613 |
| Traditionalism | -0.026 | 0.049 | -0.52 | 0.607 |
| Ecological stress | -0.084 | 0.088 | -0.951 | 0.349 |

*Notes.* Tests are two-sided. *N*_observations_ = 38.

**1.11.5. NATO membership, share of military budget from GDP, and recent**

**involvement in international conflict**

Conflict misalignments might not only be affected by historical events such as involvement in past international conflict, but also by current international dynamics. To probe this possibility, we retrieved three relevant indicators: NATO membership in 2021 (yes vs no; www.nato.int/cps/en/natohq/nato_countries.htm), share of the GDP invested in military expense (World Databank, https://data.worldbank.org/indicator/MS.MIL.XPND.GD.ZS) and very recent involvement in international conflict (year > 2000, correlates of war, 11). We then computed the same models of Table S5 and S6, adding each of these new indicators independently. We found that results remain the same for all models. Institutional quality remains a main predictor of competition by country *x*, while GDP and historical reputation remain main predictors of defense against country *x*. The share of GDP dedicated to military expenses was also significantly associated with competition against foreigners, with larger shares predicting stronger competition.

**Table S39.** Regression model of gross domestic product, institutional quality, reputation of conflict, traditionalism, and ecological stress, and share of GDP spent in military, predicting defense against country *x*.

| **Defense against country *x*** | ***b*** | ***SE*** | ***t*** | ***p*** |
| --- | --- | --- | --- | --- |
| Gross domestic product | 0.114 | 0.034 | 3.31 | 0.002 |
| Quality of institutions | 0.028 | 0.023 | 1.225 | 0.228 |
| Reputation of conflict | 0.104 | 0.018 | 5.768 | <.001 |
| Ecological stress | 0.001 | 0.024 | 0.013 | 0.99 |
| Traditionalism | -0.016 | 0.024 | -0.692 | 0.493 |
| Share GDP military | 0.008 | 0.011 | 0.701 | 0.487 |

*Notes.* Tests are two-sided. *N*_observations_ = 47.

**Table S40.** Regression model of gross domestic product, institutional quality, reputation of conflict, traditionalism, and ecological stress, and share of GDP spent in military, predicting competition by country *x*.

| **Competition by country *x*** | ***b*** | ***SE*** | ***t*** | ***p*** |
| --- | --- | --- | --- | --- |
| Gross domestic product | -0.047 | 0.088 | -0.534 | 0.596 |
| Quality of institutions | -0.117 | 0.058 | -2.008 | 0.051 |
| Reputation of conflict | -0.036 | 0.046 | -0.779 | 0.441 |
| Ecological stress | 0.014 | 0.061 | 0.23 | 0.819 |
| Traditionalism | 0.006 | 0.061 | 0.093 | 0.927 |
| Share GDP military | 0.071 | 0.029 | 2.502 | 0.017 |

*Notes.* Tests are two-sided. *N*_observations_ = 47.

**Table S41.** Regression model of gross domestic product, institutional quality, reputation of conflict, traditionalism, and ecological stress, and recent reputation of conflict (>2000), predicting defense against country *x*.

| **Defense against country *x*** | ***b*** | ***SE*** | ***t*** | ***p*** |
| --- | --- | --- | --- | --- |
| Gross domestic product | 0.124 | 0.032 | 3.927 | <.001 |
| Quality of institutions | 0.015 | 0.02 | 0.742 | 0.462 |
| Reputation of conflict | 0.126 | 0.028 | 4.517 | <.001 |
| Ecological stress | -0.004 | 0.021 | -0.191 | 0.85 |
| Traditionalism | -0.01 | 0.022 | -0.448 | 0.656 |
| Reputation of conflict (>2000) | -0.003 | 0.003 | -0.94 | 0.353 |

*Notes.* Tests are two-sided. *N*_observations_ = 49.

**Table S42.** Regression model of gross domestic product, institutional quality, reputation of conflict, traditionalism, and ecological stress, and recent reputation of conflict (>2000), predicting competition by country *x*.

| **Competition by country *x*** | ***b*** | ***SE*** | ***t*** | ***p*** |
| --- | --- | --- | --- | --- |
| Gross domestic product | -0.013 | 0.091 | -0.143 | 0.887 |
| Quality of institutions | -0.14 | 0.057 | -2.451 | 0.018 |
| Reputation of conflict | 0.011 | 0.08 | 0.141 | 0.888 |
| Ecological stress | 0.048 | 0.06 | 0.803 | 0.426 |
| Traditionalism | 0.005 | 0.064 | 0.085 | 0.933 |
| Reputation of conflict (>2000) | -0.004 | 0.01 | -0.46 | 0.648 |

*Notes.* Tests are two-sided. *N*_observations_ = 49.

**Table S43.** Regression model of gross domestic product, institutional quality, reputation of conflict, traditionalism, and ecological stress, and NATO membership, predicting defense against country *x*.

| **Defense against country *x*** | ***b*** | ***SE*** | ***t*** | ***p*** |
| --- | --- | --- | --- | --- |
| Gross domestic product | 0.143 | 0.033 | 4.298 | <.001 |
| Quality of institutions | 0.01 | 0.021 | 0.481 | 0.633 |
| Reputation of conflict | 0.1 | 0.017 | 5.807 | <.001 |
| Ecological stress | 0.009 | 0.022 | 0.4 | 0.691 |
| Traditionalism | -0.023 | 0.022 | -1.046 | 0.301 |
| Nato membership (Yes vs No) | 0.031 | 0.039 | 0.795 | 0.431 |

*Notes.* Tests are two-sided. *N*_observations_ = 51.

**Table S44.** Regression model of gross domestic product, institutional quality, reputation of conflict, traditionalism, and ecological stress, and NATO membership, predicting competition by country *x*.

| **Competition by country *x*** | ***b*** | ***SE*** | ***t*** | ***p*** |
| --- | --- | --- | --- | --- |
| Gross domestic product | -0.038 | 0.09 | -0.419 | 0.677 |
| Quality of institutions | -0.124 | 0.057 | -2.194 | 0.034 |
| Reputation of conflict | -0.008 | 0.047 | -0.178 | 0.859 |
| Ecological stress | 0.017 | 0.06 | 0.285 | 0.777 |
| Traditionalism | -0.002 | 0.059 | -0.033 | 0.974 |
| Nato membership (Yes vs No) | -0.105 | 0.106 | -0.994 | 0.326 |

*Notes.* Tests are two-sided. *N*_observations_ = 51.

**1.11.6. Other potential cross-societal factors**

In the previous sections, we showed that between-country differences in competition by country *x* and defense against country *x* were associated with relevant cross-societal factors such as the quality of institutions, GDP, and historical involvement in international conflict. There may be other relevant indicators that can contribute to conflict misperceptions. This might be particularly true for between-country differences in competition against foreigners. In fact, while the model with GDP and historical reputation explains 73% of the variance, the model with quality of institutions explains only 46% of variance (see Table Section 1.3). Therefore, in this section, we considered other potential factors, such as deep historical institutional measures (accumulated state experience (12), and kin-based institutions, (13), prosocial measures such as trust and cooperation (14, 15), or years of being colonized (16). To avoid problems with overfitting and addition of too many controls (17, 18), we first consider bivariate correlations. As shown in Fig. S8, cooperation, trust, years of colonization and the presence of state are neither associated with competition by country *x*, nor defense against country *x.*

The kinship intensity index was associated with both competition and defense. To further explore its role, we then ran two country-level regressions (for competition and defense), controlling for the other significant factors such as quality of institutions, historical reputation of conflict, and GDP. While we found that the effect of kinship institutions became insignificant in the model predicting defense against country *x* (Table S45), it remained significant when predicting between-country differences in competition against foreigners (Table S46). This results suggest that not only the quality of institutions (democracy, rule of law, governments effectiveness), but also their deep historical roots might be associated with how people differ in competing against foreigners around the globe.

**
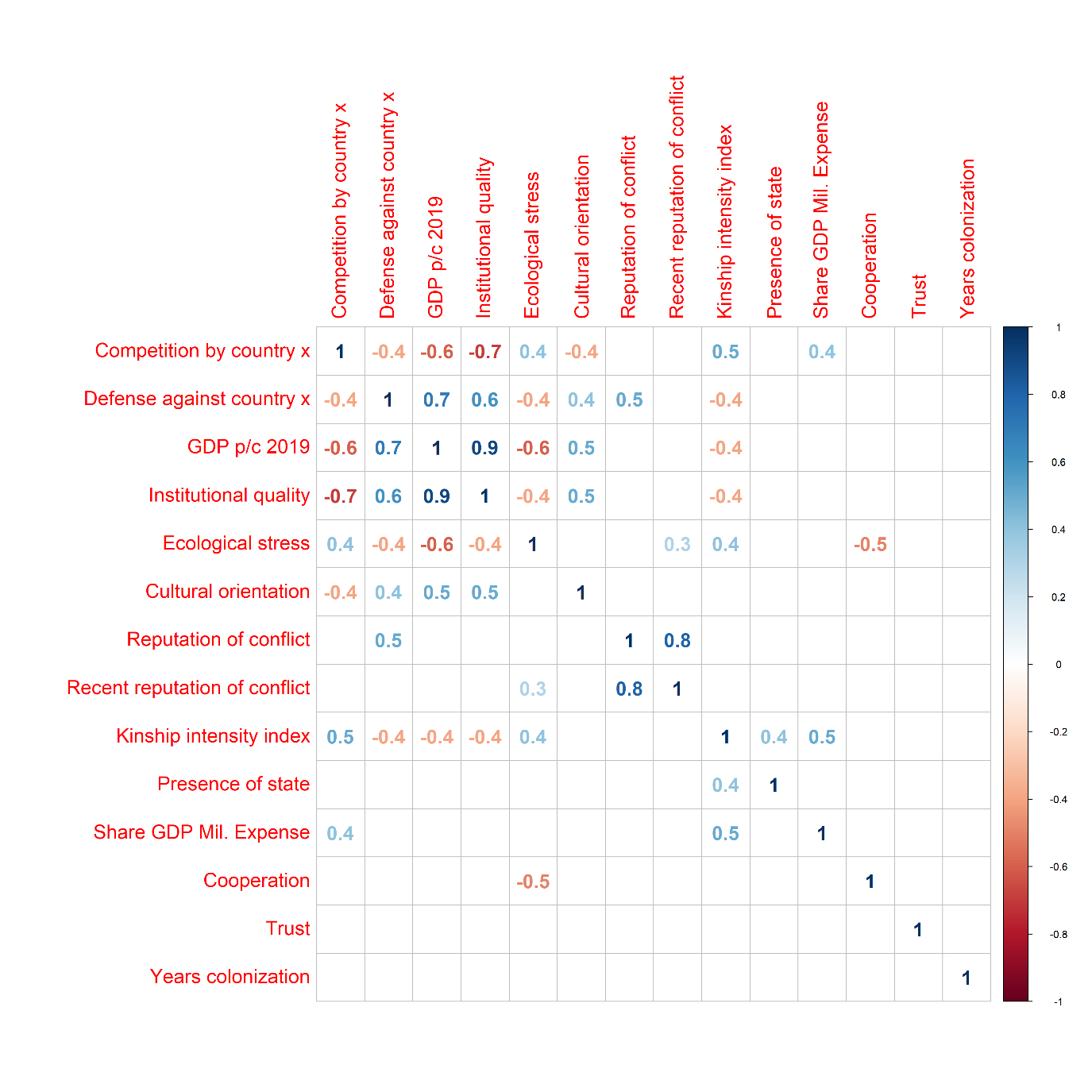
**

**Fig S8. Bivariate correlations among cross-societal indicators.** Only significant correlations are shown.

**Table S45.** Regression model of gross domestic product, institutional quality, reputation of conflict, kinship intensity index, predicting defense against country *x*.

| **Defense against country *x*** | ***b*** | ***SE*** | ***t*** | ***p*** |
| --- | --- | --- | --- | --- |
| Gross domestic product | 0.147 | 0.029 | 5.049 | <.001 |
| Quality of institutions | 0.001 | 0.019 | 0.079 | 0.938 |
| Reputation of conflict | 0.094 | 0.016 | 5.902 | <.001 |
| Kinship intensity index | -0.002 | 0.019 | -0.132 | 0.895 |

*Notes.* Tests are two-sided. *N*_observations_ = 50.

**Table S46.** Regression model of gross domestic product, institutional quality, reputation of conflict, and kinship intensity index predicting competition by country *x*.

| **Competition by country *x*** | ***b*** | ***SE*** | ***t*** | ***p*** |
| --- | --- | --- | --- | --- |
| Gross domestic product | -0.058 | 0.075 | -0.775 | 0.442 |
| Quality of institutions | -0.100 | 0.048 | -2.058 | 0.045 |
| Reputation of conflict | 0.008 | 0.041 | 0.193 | 0.848 |
| Kinship intensity index | 0.119 | 0.048 | 2.454 | 0.018 |

*Notes.* Tests are two-sided. *N*_observations_ = 50.

**1.12.** **Conditional defense across different GDP levels**

Another potential concern is that findings related to reputation of conflict and GDP are driven by a particular set of rich or poor countries. In this section, we split the sample based on low, medium, and high GDP 2019 per capita to test whether our results hold across different wealth levels. In Table S47 to Table S49, we test whether GDP and reputation of conflict are consistently predicting conditional investment to conflict across different wealth levels. Results show the same results across countries with low, medium, and high GDP.

**Table S47.** Mixed effect models with cross-societal indicators of opponents predicting defense in conflict from participants belonging to countries with lower gross domestic product per capita.

| **Defense - low gdp** | ***b*** | ***SE*** | ***t*** | ***p*** |
| --- | --- | --- | --- | --- |
| Gross domestic product - opponent | 0.079 | 0.028 | 2.885 | 0.006 |
| Traditionalism - opponent | -0.013 | 0.019 | -0.663 | 0.511 |
| Institutional quality - opponent | 0.011 | 0.018 | 0.627 | 0.534 |
| Ecological stress - opponent | -0.003 | 0.016 | -0.155 | 0.877 |
| Reputation of conflict - opponent | 0.074 | 0.014 | 5.212 | <0.001 |
| *N_countries_* | 12 |  |  |  |

*Notes.* Tests are two-sided. *N*_observations_ = 72,381; *N*_subjects_ = 2953; *N*_country-opponent_ = 51.

**Table S48.** Mixed effect models with cross-societal indicators of opponents predicting investment in conflict from participants belonging to countries with medium gross domestic product per capita.

| **Defense - medium gdp** | ***b*** | ***SE*** | ***t*** | ***p*** |
| --- | --- | --- | --- | --- |
| Gross domestic product - opponent | 0.127 | 0.032 | 3.969 | <0.001 |
| Traditionalism - opponent | -0.003 | 0.022 | -0.141 | 0.889 |
| Institutional quality - opponent | 0.017 | 0.021 | 0.824 | 0.415 |
| Ecological stress - opponent | -0.015 | 0.019 | -0.781 | 0.439 |
| Reputation of conflict - opponent | 0.111 | 0.017 | 6.699 | <0.001 |
| *N_countries_* | 26 |  |  |  |

*Notes.* Tests are two-sided. *N*_observations_ = 162,032; *N*_subjects_ = 6610; *N*_country-opponent_ = 51.

**Table S49.** Mixed effect models with cross-societal indicators of opponents predicting investment in conflict from participants belonging to countries with higher gross domestic product per capita.

| **Defense - high gdp** | ***b*** | ***SE*** | ***t*** | ***p*** |
| --- | --- | --- | --- | --- |
| Gross domestic product - opponent | 0.153 | 0.032 | 4.818 | <0.001 |
| Traditionalism - opponent | -0.009 | 0.022 | -0.432 | 0.668 |
| Institutional quality - opponent | -0.003 | 0.02 | -0.169 | 0.867 |
| Ecological stress - opponent | -0.022 | 0.019 | -1.179 | 0.245 |
| Reputation of conflict - opponent | 0.117 | 0.016 | 7.115 | <0.001 |
| *N_countries_* | 13 |  |  |  |

*Notes.* Tests are two-sided. *N*_observations_ = 80,861; *N*_subjects_ = 3300; *N*_country-opponent_ = 51.

**1.13. Bilateral distances**

In this section we report the bilateral distance analyses predicting absolute differences in misalignments. In particular, we assesses whether bilateral socio-psychological distances (19), geographical distances (20), bilateral trade (11), genetic distance (21), and migration flow (<https://databank.worldbank.org/source/global-bilateral-migration>), predict the extent of absolute misalignments between countries. Socio-psychological distance (or cultural distance) is a measure of the overall psychological differences between nations. To build this index, we retrieved bilateral cultural distances data from <http://culturaldistance.muth.io/> (all dimensions).This indicator is calculated from data on beliefs, values and behaviors that people have about their own nation retrieved from the world value survey (two waves: 2005-2009; 2010-2014; for a complete report of the analytic strategy to calculate this indicator see (19)). The world value survey dataset is composed of 170,247 participants from 80 nations (which altogether covers 85% of the world population). The list of measures used to calculate the socio-psychological distances can be found here: <https://michael.muthukrishna.com/cultural-distance-data/>. Geographical bilateral distances measure city-level data to account for the geographic distribution of population inside each nation (20). Geographical distance is available for 225 countries, and consists of the distance between two countries based on bilateral distances between the biggest cities of those two countries. Bilateral trade measures the trade flows between states (as defined by the Correlates of War project (11) for the period 1870-2014). The data include information on both bilateral trade flows and total national imports and exports. Genetic distance, a measure associated with the amount of time elapsed since two populations’ last common ancestors, is retrieved from Spolaore and Wacziarg. The dataset covers 647 microsatellite loci and 267 populations. Spolaore and Wacziarg linked these population-level genetic distances to countries using ethnic composition data from Fearon. To assess migration flow, we retrieved data from the global bilateral migration of the world bank (<https://databank.worldbank.org/source/global-bilateral-migration>).

We find that socio-psychological distance is the only significant predictor, suggesting that distance between the two countries based on their values, attitudes and behaviors (or more broadly, institutions), is associated with higher absolute misperceptions in conflict (Table S50).

**Table S50.** Average bilateral trade, absolute bilateral cultural distance, genetic distance, geographical distance, and bilateral migration flow predicting absolute conflict misalignments between pair of countries

| **Bilateral misalignments** | ***b*** | ***SE*** | ***t*** | ***p*** |
| --- | --- | --- | --- | --- |
| Trade | -0.008 | 0.013 | -0.585 | 0.559 |
| Socio-psychological distance | 1.933 | 0.218 | 8.862 | <0.001 |
| Genetic distance | -0.415 | 1.332 | -0.312 | 0.755 |
| Migration flow | 0.006 | 0.011 | 0.546 | 0.585 |
| Geographical distance | -0.017 | 0.019 | -0.893 | 0.372 |

*Notes.* Tests are two-sided. *N*_observations_ = 619; *N*_countries_ = 37.

**1.14. Misalignment within vs. between countries**

In this section, we show the difference in absolute misalignments between countries and within countries (Fig. S9), and a stacked bar plot showing these differences by country (Fig. S10). Both plots show that the extent of conflict misalignments is higher between countries than within countries and that such difference is wide-spread around the world.

**
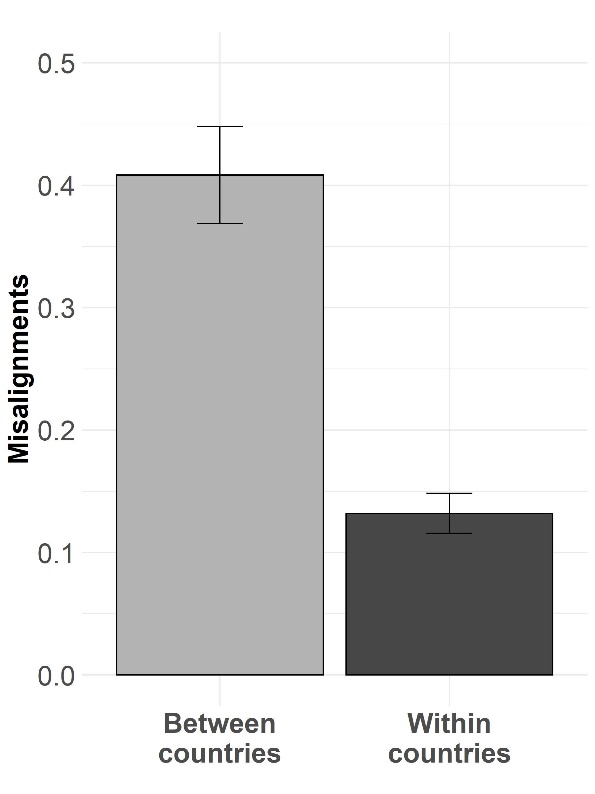
**

Figure S9. **Absolute conflict misalignments within and between countries.** Bar chart of the absolute difference between defense against an outgroup country and the actual competition of that country against foreigners (between country misalignment), and the absolute difference between defense against people from their own country, and competition toward people of their own country (within country misalignment).

**
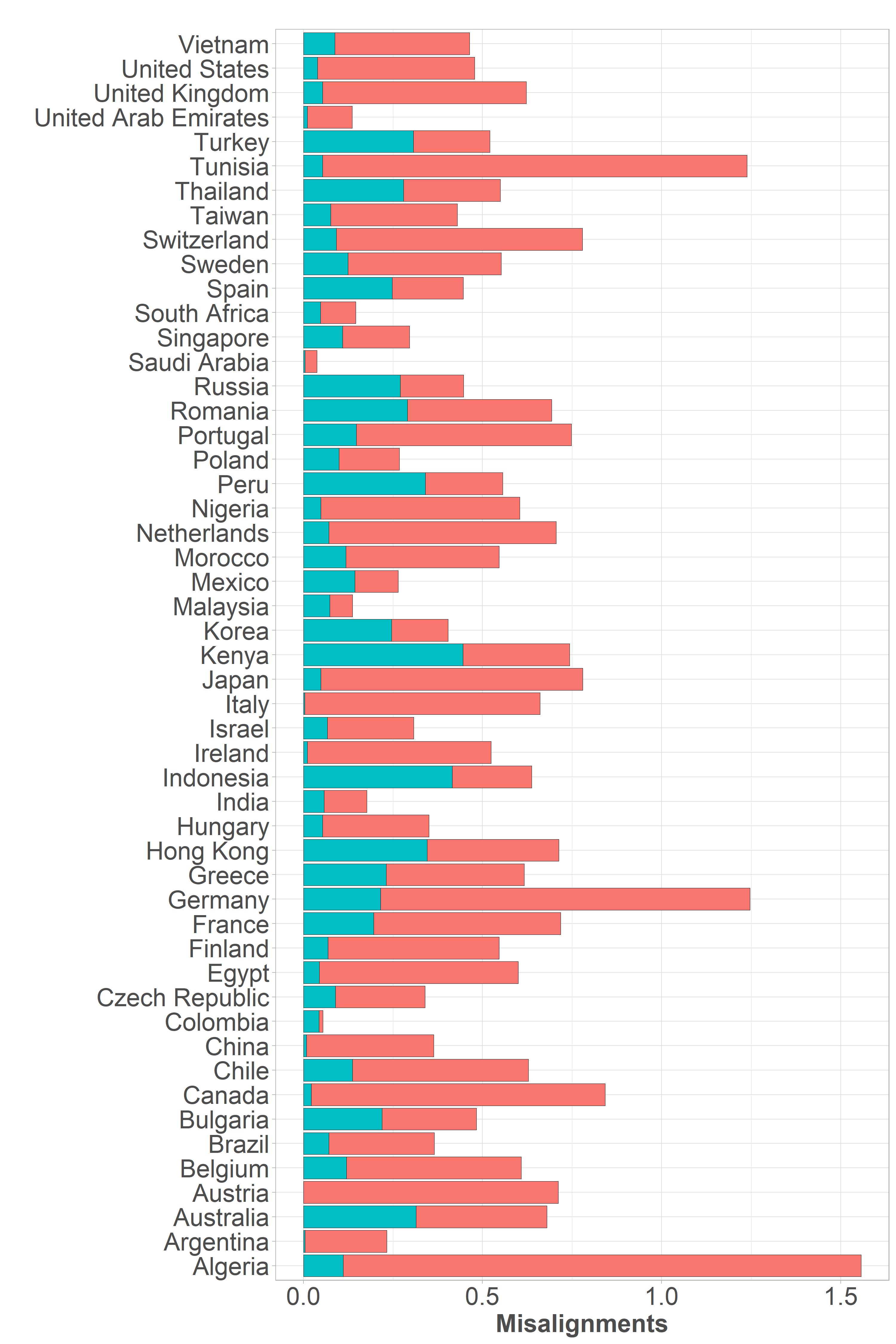
**

Figure S10. **Stacked bar plot of conflict misalignments.** Differences in conflict misalignments between countries and within countries. Red bars show the absolute value of conflict misalignments between countries. Blue bars show the absolute value of conflict misalignments within countries

1. **Descriptives**

**Table S51.** Sample descriptives.

| ***Country*** | ***Country iso 3*** | | ***N*** | ***% Women*** | ***M_age_ (SD)*** | ***Language*** |
| --- | --- | --- | --- | --- | --- | --- |
| Algeria | DZA | 198 | | 29.29 | 36.77 (10.76) | Arabic |
| Argentina | ARG | 231 | | 55.41 | 37.21 (12.52) | Spanish |
| Australia | AUS | 254 | | 53.94 | 44.18 (12.71) | English |
| Austria | AUT | 254 | | 52.36 | 39.39 (13.38) | German |
| Belgium | BEL | 232 | | 52.16 | 41.25 (13.16) | French & Dutch |
| Brazil | BRA | 253 | | 50.99 | 37.72 (12.26) | Portuguese |
| Bulgaria | BGR | 251 | | 43.03 | 38.73 (11.96) | Bulgarian |
| Canada | CAN | 277 | | 53.79 | 44.68 (12.72) | French & English |
| Chile | CHL | 234 | | 62.82 | 34.49 (12.06) | Spanish |
| China | CHN | 244 | | 47.54 | 37.77 (11.34) | Simplified Chinese |
| Colombia | COL | 231 | | 50.22 | 38.73 (12.77) | Spanish |
| Czechia | CZE | 257 | | 50.97 | 39.41 (13.65) | Czech |
| Egypt | EGY | 247 | | 41.30 | 35.08 (11.03) | Arabic |
| Finland | FIN | 276 | | 51.81 | 41.69 (12.72) | Finnish |
| France | FRA | 234 | | 52.56 | 43.66 (11.88) | French |
| Germany | DEU | 232 | | 51.95 | 45.54 (12.35) | German |
| Greece | GRC | 229 | | 54.15 | 38.42 (12.17) | Greek |
| Hong Kong | HKG | 252 | | 50.00 | 38.69 (12.47) | English & Chinese |
| Hungary | HUN | 260 | | 51.92 | 40.84 (14.29) | Hungarian |
| India | IND | 225 | | 50.22 | 37.65 (12.41) | English |
| Indonesia | IDN | 231 | | 48.05 | 37.90 (11.89) | Indonesian |
| Ireland | IRL | 266 | | 57.14 | 40.67 (11.76) | English |
| Israel | ISR | 254 | | 47.03 | 40.07 (13.11) | Hebrew |
| Italy | ITA | 258 | | 50.78 | 40.91 (12.98) | Italian |
| Japan | JPN | 226 | | 39.38 | 42.98 (11.18) | Japanese |
| Kenya | KEN | 249 | | 58.23 | 31.38 (9.32) | English |
| Korea | KOR | 260 | | 45.00 | 40.59 (11.85) | Korean |
| Malaysia | MYS | 258 | | 45.35 | 37.28 (11.98) | English & Malay |
| Mexico | MEX | 250 | | 50.80 | 37.57 (12.01) | Spanish |
| Morocco | MAR | 253 | | 35.17 | 30.77 (9.39) | Arabic |
| Netherlands | NLD | 239 | | 53.56 | 42.5 (12.51) | Dutch |
| Nigeria | NGA | 226 | | 71.24 | 30.3 (10.63) | English |
| Peru | PER | 267 | | 52.43 | 34.09 (11.11) | Spanish |
| Poland | POL | 252 | | 48.81 | 38.45 (13.05) | Polish |
| Portugal | PRT | 271 | | 51.29 | 40.25 (13.16) | Portuguese |
| Romania | ROU | 258 | | 49.22 | 39.41 (13.51) | Romanian |
| Russia | RUS | 237 | | 51.05 | 40.77 (12.43) | Russian |
| S. Arabia | SAU | 236 | | 47.88 | 34.03 (9.76) | Arabic & English |
| Singapore | SGP | 265 | | 47.55 | 39.78 (12.53) | English |
| S. Africa | ZAF | 253 | | 52.57 | 37.48 (12.86) | English |
| Spain | ESP | 254 | | 46.46 | 40.45 (12.22) | Spanish |
| Sweden | SWE | 241 | | 50.21 | 43.59 (13.26) | Swedish |
| Switzerland | CHE | 283 | | 53.71 | 41.59 (12.97) | German & French |
| Taiwan | TWN | 290 | | 46.55 | 36.88 (12.06) | Chinese |
| Thailand | THA | 311 | | 50.16 | 39.84 (13.02) | Thai |
| Tunisia | TUN | 299 | | 39.46 | 40.51 (11.91) | French & Arabic |
| Turkey | TUR | 270 | | 52.96 | 35.33 (11.41) | Turkish |
| UAE | ARE | 270 | | 46.67 | 34.24 (10.28) | Arabic & English |
| UK | GBR | 262 | | 53.82 | 43.02 (13.29) | English |
| USA | USA | 229 | | 51.53 | 44.00 (14.04) | English |
| Vietnam | VNM | 274 | | 51.46 | 33.42 (9.68) | Vietnamese |
| **Total** |  | **12.863** | | **50.04** | **38.86 (12.29)** |  |

1. **Game theory**


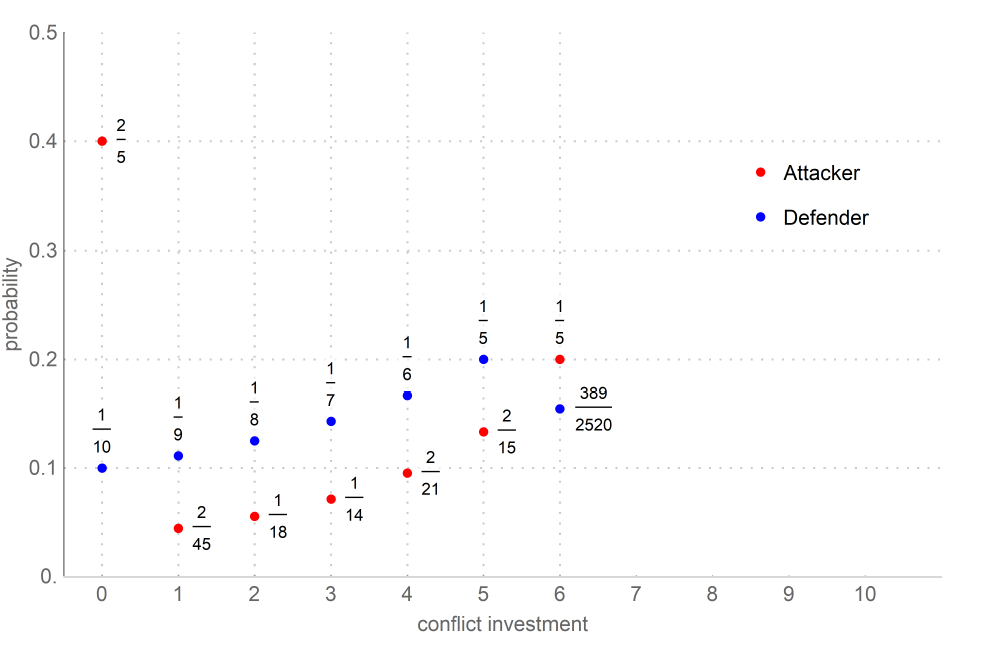


Figure S11. **Equilibrium strategies for equal endowments.** Figure from Meder et al. (22) on expected probabilities of attack and defense investment for each monetary units.

**3.1 Conflict misalignments and conflict misperceptions**

In the previous sections, we show consistent evidence of conflict misalignments (e.g., Figure S9 and S10), and that such conflict misalignments are systematically associated with cross-country differences in institutions, wealth, and historical involvement in international conflict (e.g., section 1.3). Investments in defense against a country *x* and competition by country *x* are not in line with expected rational investments (Fig. S12). How to interpret such misalignments then? We claim that conflict misalignments point to shared conflict misperceptions between citizens and foreigners. Conflict misperceptions can be defined by a discrepancy between people’s beliefs about a certain opponent’s behavior and the actual behavior of that opponent (23). In the attacker-defender game, beliefs about the investments of the opponents are particularly important for defense investments, as defenders lose their resources if they invest less than their attackers.

We have four reasons to believe that conflict misalignments point to shared conflict misperceptions. First, defense investments were significantly associated with the country of the opponent (mixed-effects model of country of the opponent predicting investments in defense; *F(*315,535*)* = 90.956, *p* < .001), suggesting that individuals used the country of the opponent as information to condition their investments. Second, the extent of conflict misalignments was lower within countries, than between countries. This suggest that, while defenders were fairly accurate in anticipating their fellow citizens’ competition, they failed to do so when interacting with opponents of different countries (see Figure S9 and S10). Third, we found that conflict misalignments were systematically associated to prominent cross-societal indicators such as wealth, historical involvements conflict and institutions. Finally, these results are in line with previous cross-cultural research on international cooperation that explicitly measured beliefs. In line with our results, this past research found that cooperation beliefs about a country’s opponent were negatively associated with the actual cooperation of opponents of that country (23).

**
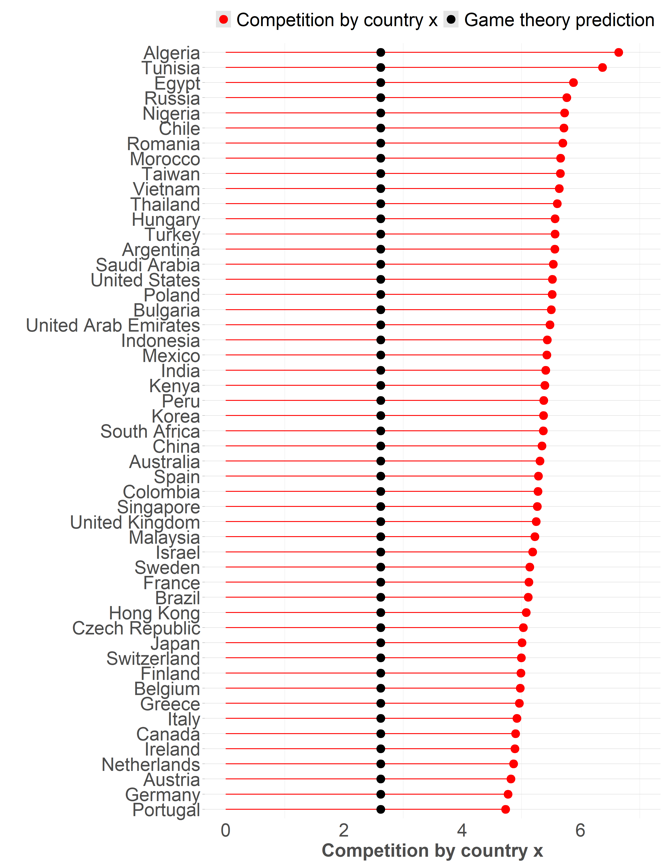

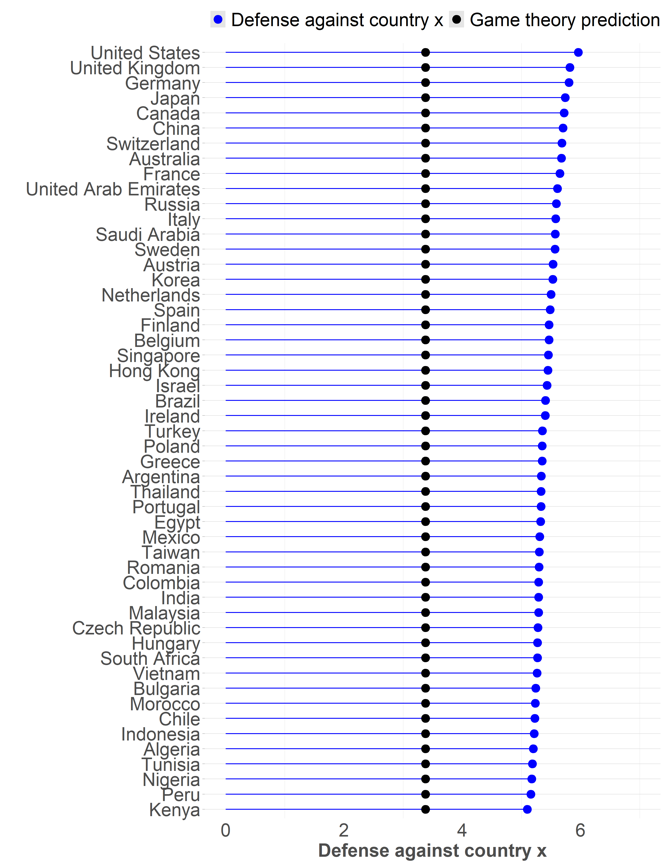
**

Figure S12. **Expected rational investments, competition by country x, and defense by country x.**

**References**

1. C. K. W. De Dreu, J. Gross, Revisiting the Form and Function of Conflict: Neurobiological, Psychological and Cultural Mechanisms for Attack and Defense Within and between Groups. *Behav. Brain Sci.* **42**, e116 (2018).

2. C. K. W. De Dreu, R. Pliskin, M. Rojek-Giffin, Z. Méder, J. Gross, Political games of attack and defence. *Philos. Trans. R. Soc. B Biol. Sci.* **376**, 20200135 (2021).

3. Z. Zhang, Multiple imputation with multivariate imputation by chained equation (MICE) package. *Ann. Transl. Med.* **4**, 30 (2016).

4. D. J. Stekhoven, Using the missForest Package. *R package* (2011).

5. G. James, D. Witten, T. Hastie, R. Tibshirani, An Introduction to Statistical Learning. New York: Springer (2013).

6. R. Inglehart, W. E. Baker, Modernization, cultural change, and the persistence of traditional values. *Am. Sociol. Rev.* **65**, 19–51 (2000).

7. S. Huntington, The clash of civilizations? *Foreign Aff.* **72**, 22–49 (1993).

8. D. R. Murray, M. Schaller, Historical Prevalence of Infectious Diseases Within 230 Geopolitical Regions: A Tool for Investigating Origins of Culture: *J. Cross. Cult. Psychol.* **41**, 99–108 (2009).

9. A. Maddison, The Maddison-Project. *cefia.aks.ac.kr* (2013).

10. N. Coppedge, Michael, John Gerring, Carl Henrik Knutsen, Staffan I. Lindberg, Jan Teorell, *et al.*, VDem 2021 Dataset v12 (2022).

11. Correlates of War Project, “State System Membership List, v2016.” *Online* (2017).

12. O. Borcan, O. Olsson, L. Putterman, State history and economic development: evidence from six millennia. *J. Econ. Growth* **23**, 1–40 (2018).

13. J. F. Schulz, D. Bahrami-Rad, J. P. Beauchamp, J. Henrich, The Church, intensive kinship, and global psychological variation. *Science.* **366** (2019).

14. A. Romano, M. Sutter, J. H. Liu, T. Yamagishi, D. Balliet, National parochialism is ubiquitous across 42 nations around the world. *Nat. Commun.* **12**, 1–8 (2021).

15. A. Falk, *et al.*, Global Evidence on Economic Preferences. *Q. J. Econ.* **133**, 1645–1692 (2018).

16. P. Ziltener, D. Künzler, A. Walter, Research Note: Measuring the Impacts of Colonialism: A New Data Set for the Countries of Africa and Asia. *J. World-Systems Res.* **23**, 156–190 (2017).

17. A. C. Wysocki, K. M. Lawson, M. Rhemtulla, Statistical Control Requires Causal Justification. *Adv. Methods Pract. Psychol. Sci.* **5**, 1–19 (2022).

18. P. Peduzzi, J. Concato, E. Kemper, T. R. Holford, A. R. Feinstem, A simulation study of the number of events per variable in logistic regression analysis. *J. Clin. Epidemiol.* **49**, 1373–1379 (1996).

19. M. Muthukrishna, *et al.*, Beyond Western, Educated, Industrial, Rich, and Democratic (WEIRD) Psychology: Measuring and Mapping Scales of Cultural and Psychological Distance. *Psychol. Sci.* **31**, 678–701 (2020).

20. T. Mayer, S. Zignago, CEPII - Notes on CEPII’s distances measures: The GeoDist database. *CEPII Work. Pap.* (2011) (November 22, 2021).

21. E. Spolaore, R. Wacziarg, War and Relatedness. *Rev. Econ. Stat.* **98**, 925–939 (2016).

22. Z. Z. Méder, C. K. W. De Dreu, J. Gross, Equilibria of Attacker-Defender Games (2022).

23. A. R. Dorrough, A. Glöckner, Multinational investigation of crosssocietal cooperation. *Proc. Natl. Acad. Sci. U. S. A.* **113**, 10836–10841 (2016).
